# Supplementary material for: The biosynthesis of phospholipids is linked to the cell cycle in a model eukaryote
Source: Biochim Biophys Acta Mol Cell Biol Lipids. 2021 Aug;1866(8):158965. doi: 10.1016/j.bbalip.2021.158965 (PMC8202326; doi:10.1016/j.bbalip.2021.158965)
Supplement: Supplementary file 1 — Supplementary Figures and Tables for Vitova et al. 'The biosynthesis of phospholipids is linked to the cell cycle in a model eukaryote'. [file mmc1.docx]

**Supplementary Information**

For Vítová *et al.*, The biosynthesis of phospholipids is linked to the cell cycle in a model eukaryote.

|  | PC | | | | | | |  | PI | | | | | | |
| --- | --- | --- | --- | --- | --- | --- | --- | --- | --- | --- | --- | --- | --- | --- | --- |
|  | +2h | +4h | +6h | +8h | +10h | +13h | +15h |  | +2h | +4h | +6h | +8h | +10h | +13h | +15h |
| 12:0/16:3 |  |  |  |  |  |  |  |  |  |  |  |  |  |  |  |
| 12:0/18:3 |  |  |  |  |  |  |  |  |  |  |  |  |  |  |  |
| 12:0/22:1 |  |  |  |  |  |  |  |  |  |  |  |  |  |  |  |
| 12:1/18:3 |  |  |  |  |  |  |  |  |  |  |  |  |  |  |  |
| 14:0/20:4 |  |  |  |  |  |  |  |  |  |  |  |  |  |  |  |
| 14:0/22:1 |  |  |  |  |  |  |  |  |  |  |  |  |  |  |  |
| 14:0/24:4 |  |  |  |  |  |  |  |  |  |  |  |  |  |  |  |
| 14:1/22:1 |  |  |  |  |  |  |  |  |  |  |  |  |  |  |  |
| 14:1/22:2 |  |  |  |  |  |  |  |  |  |  |  |  |  |  |  |
| 14:2/20:1 |  |  |  |  |  |  |  |  |  |  |  |  |  |  |  |
| 14:2/20:3 |  |  |  |  |  |  |  |  |  |  |  |  |  |  |  |
| 16:0/16:0 |  |  |  |  |  |  |  |  |  |  |  |  |  |  |  |
| 16:0/16:1 |  |  |  |  |  |  |  |  |  |  |  |  |  |  |  |
| 16:0/16:2 |  |  |  |  |  |  |  |  |  |  |  |  |  |  |  |
| 16:0/16:3 |  |  |  |  |  |  |  |  |  |  |  |  |  |  |  |
| 16:0/16:4 |  |  |  |  |  |  |  |  |  |  |  |  |  |  |  |
| 16:0/18:1 |  |  |  |  |  |  |  |  |  |  |  |  |  |  |  |
| 16:0/18:2 |  |  |  |  |  |  |  |  |  |  |  |  |  |  |  |
| 16:0/18:3 |  |  |  |  |  |  |  |  |  |  |  |  |  |  |  |
| 16:0/18:4 |  |  |  |  |  |  |  |  |  |  |  |  |  |  |  |
| 16:0/20:3 |  |  |  |  |  |  |  |  |  |  |  |  |  |  |  |
| 16:0/20:4 |  |  |  |  |  |  |  |  |  |  |  |  |  |  |  |
| 16:0/22:1 |  |  |  |  |  |  |  |  |  |  |  |  |  |  |  |
| 16:0/22:3 |  |  |  |  |  |  |  |  |  |  |  |  |  |  |  |
| 16:0/22:4 |  |  |  |  |  |  |  |  |  |  |  |  |  |  |  |
| 16:0/24:0 |  |  |  |  |  |  |  |  |  |  |  |  |  |  |  |
| 16:0/24:1 |  |  |  |  |  |  |  |  |  |  |  |  |  |  |  |
| 16:0/24:2 |  |  |  |  |  |  |  |  |  |  |  |  |  |  |  |
| 16:0/24:3 |  |  |  |  |  |  |  |  |  |  |  |  |  |  |  |
| 16:0/24:4 |  |  |  |  |  |  |  |  |  |  |  |  |  |  |  |
| 16:1/18:1 |  |  |  |  |  |  |  |  |  |  |  |  |  |  |  |
| 16:1/18:1 |  |  |  |  |  |  |  |  |  |  |  |  |  |  |  |
| 16:1/18:2 |  |  |  |  |  |  |  |  |  |  |  |  |  |  |  |
| 16:1/18:3 |  |  |  |  |  |  |  |  |  |  |  |  |  |  |  |
| 16:1/18:4 |  |  |  |  |  |  |  |  |  |  |  |  |  |  |  |
| 16:1/20:0 |  |  |  |  |  |  |  |  |  |  |  |  |  |  |  |
| 16:1/20:1 |  |  |  |  |  |  |  |  |  |  |  |  |  |  |  |
| 16:1/24:4 |  |  |  |  |  |  |  |  |  |  |  |  |  |  |  |
| 16:2/16:0 |  |  |  |  |  |  |  |  |  |  |  |  |  |  |  |
| 16:2/18:1 |  |  |  |  |  |  |  |  |  |  |  |  |  |  |  |
| 16:2/18:2 |  |  |  |  |  |  |  |  |  |  |  |  |  |  |  |
| 16:2/18:3 |  |  |  |  |  |  |  |  |  |  |  |  |  |  |  |
| 16:2/24:4 |  |  |  |  |  |  |  |  |  |  |  |  |  |  |  |
| 16:3/16:3 |  |  |  |  |  |  |  |  |  |  |  |  |  |  |  |
| 16:3/16:4 |  |  |  |  |  |  |  |  |  |  |  |  |  |  |  |
| 16:3/18:1 |  |  |  |  |  |  |  |  |  |  |  |  |  |  |  |
| 16:3/18:2 |  |  |  |  |  |  |  |  |  |  |  |  |  |  |  |
| 16:3/18:3 |  |  |  |  |  |  |  |  |  |  |  |  |  |  |  |
| 16:3/18:4 |  |  |  |  |  |  |  |  |  |  |  |  |  |  |  |
| 16:3/22:1 |  |  |  |  |  |  |  |  |  |  |  |  |  |  |  |
| 16:3/24:2 |  |  |  |  |  |  |  |  |  |  |  |  |  |  |  |
| 16:3/24:3 |  |  |  |  |  |  |  |  |  |  |  |  |  |  |  |
| 16:3/24:4 |  |  |  |  |  |  |  |  |  |  |  |  |  |  |  |

|  | PC | | | | | | |  | PI | | | | | | |
| --- | --- | --- | --- | --- | --- | --- | --- | --- | --- | --- | --- | --- | --- | --- | --- |
|  | +2h | +4h | +6h | +8h | +10h | +13h | +15h |  | +2h | +4h | +6h | +8h | +10h | +13h | +15h |
| 16:4/16:0 |  |  |  |  |  |  |  |  |  |  |  |  |  |  |  |
| 16:4/16:4 |  |  |  |  |  |  |  |  |  |  |  |  |  |  |  |
| 16:4/18:1 |  |  |  |  |  |  |  |  |  |  |  |  |  |  |  |
| 16:4/18:2 |  |  |  |  |  |  |  |  |  |  |  |  |  |  |  |
| 16:4/18:3 |  |  |  |  |  |  |  |  |  |  |  |  |  |  |  |
| 16:4/18:4 |  |  |  |  |  |  |  |  |  |  |  |  |  |  |  |
| 16:4/20:2 |  |  |  |  |  |  |  |  |  |  |  |  |  |  |  |
| 16:4/22:1 |  |  |  |  |  |  |  |  |  |  |  |  |  |  |  |
| 16:4/24:1 |  |  |  |  |  |  |  |  |  |  |  |  |  |  |  |
| 16:4/24:4 |  |  |  |  |  |  |  |  |  |  |  |  |  |  |  |
| 18:0/22:1 |  |  |  |  |  |  |  |  |  |  |  |  |  |  |  |
| 18:1/18:1 |  |  |  |  |  |  |  |  |  |  |  |  |  |  |  |
| 18:1/18:2 |  |  |  |  |  |  |  |  |  |  |  |  |  |  |  |
| 18:1/18:3 |  |  |  |  |  |  |  |  |  |  |  |  |  |  |  |
| 18:1/18:4 |  |  |  |  |  |  |  |  |  |  |  |  |  |  |  |
| 18:1/20:1 |  |  |  |  |  |  |  |  |  |  |  |  |  |  |  |
| 18:1/22:1 |  |  |  |  |  |  |  |  |  |  |  |  |  |  |  |
| 18:1/22:4 |  |  |  |  |  |  |  |  |  |  |  |  |  |  |  |
| 18:1/24:0 |  |  |  |  |  |  |  |  |  |  |  |  |  |  |  |
| 18:1/24:1 |  |  |  |  |  |  |  |  |  |  |  |  |  |  |  |
| 18:1/24:2 |  |  |  |  |  |  |  |  |  |  |  |  |  |  |  |
| 18:1/24:3 |  |  |  |  |  |  |  |  |  |  |  |  |  |  |  |
| 18:1/24:4 |  |  |  |  |  |  |  |  |  |  |  |  |  |  |  |
| 18:2/18:1 |  |  |  |  |  |  |  |  |  |  |  |  |  |  |  |
| 18:2/18:2 |  |  |  |  |  |  |  |  |  |  |  |  |  |  |  |
| 18:2/18:3 |  |  |  |  |  |  |  |  |  |  |  |  |  |  |  |
| 18:2/18:4 |  |  |  |  |  |  |  |  |  |  |  |  |  |  |  |
| 18:2/22:1 |  |  |  |  |  |  |  |  |  |  |  |  |  |  |  |
| 18:2/22:3 |  |  |  |  |  |  |  |  |  |  |  |  |  |  |  |
| 18:2/22:4 |  |  |  |  |  |  |  |  |  |  |  |  |  |  |  |
| 18:2/24:4 |  |  |  |  |  |  |  |  |  |  |  |  |  |  |  |
| 18:3/16:0 |  |  |  |  |  |  |  |  |  |  |  |  |  |  |  |
| 18:3/16:3 |  |  |  |  |  |  |  |  |  |  |  |  |  |  |  |
| 18:3/16:4 |  |  |  |  |  |  |  |  |  |  |  |  |  |  |  |
| 18:3/18:1 |  |  |  |  |  |  |  |  |  |  |  |  |  |  |  |
| 18:3/18:2 |  |  |  |  |  |  |  |  |  |  |  |  |  |  |  |
| 18:3/18:3 |  |  |  |  |  |  |  |  |  |  |  |  |  |  |  |
| 18:3/18:4 |  |  |  |  |  |  |  |  |  |  |  |  |  |  |  |
| 18:3/20:1 |  |  |  |  |  |  |  |  |  |  |  |  |  |  |  |
| 18:3/22:1 |  |  |  |  |  |  |  |  |  |  |  |  |  |  |  |
| 18:3/22:2 |  |  |  |  |  |  |  |  |  |  |  |  |  |  |  |
| 18:3/22:3 |  |  |  |  |  |  |  |  |  |  |  |  |  |  |  |
| 18:3/22:4 |  |  |  |  |  |  |  |  |  |  |  |  |  |  |  |
| 18:3/24:0 |  |  |  |  |  |  |  |  |  |  |  |  |  |  |  |
| 18:3/24:1 |  |  |  |  |  |  |  |  |  |  |  |  |  |  |  |
| 18:3/24:2 |  |  |  |  |  |  |  |  |  |  |  |  |  |  |  |
| 18:3/24:3 |  |  |  |  |  |  |  |  |  |  |  |  |  |  |  |
| 18:3/24:4 |  |  |  |  |  |  |  |  |  |  |  |  |  |  |  |
| 18:4/18:4 |  |  |  |  |  |  |  |  |  |  |  |  |  |  |  |
| 18:4/20:0 |  |  |  |  |  |  |  |  |  |  |  |  |  |  |  |
| 18:4/22:4 |  |  |  |  |  |  |  |  |  |  |  |  |  |  |  |
| 18:4/24:4 |  |  |  |  |  |  |  |  |  |  |  |  |  |  |  |

*Figure S1. Matrix showing the frequency of the detection of isoforms of PC (left) and PI (right) in the samples used in the present study, through the cell cycle of* Desmodesmus quadricauda, *using mass spectrometry. White indicates that the isoform was not found in any of the samples, green indicates that that isoform was found in all samples tested and blue that it was found in one sample. Collection points: +2 h, early part of G_1_; +4 h, mid-G_1_ (first CP); +6 h, end of G_1_ and pS; +8 h, S and second CP; +10 h, G_2_; +13 h, M; +15 h, G_3_, for others see* Figure 1*.*

|  | PI | | | | | | |  | PIP | | | | | | |
| --- | --- | --- | --- | --- | --- | --- | --- | --- | --- | --- | --- | --- | --- | --- | --- |
|  | +2h | +4h | +6h | +8h | +10h | +13h | +15h |  | +2h | +4h | +6h | +8h | +10h | +13h | +15h |
| 12:0/16:3 |  |  |  |  |  |  |  |  |  |  |  |  |  |  |  |
| 12:0/18:3 |  |  |  |  |  |  |  |  |  |  |  |  |  |  |  |
| 12:0/22:1 |  |  |  |  |  |  |  |  |  |  |  |  |  |  |  |
| 12:1/18:3 |  |  |  |  |  |  |  |  |  |  |  |  |  |  |  |
| 14:0/22:1 |  |  |  |  |  |  |  |  |  |  |  |  |  |  |  |
| 14:0/24:4 |  |  |  |  |  |  |  |  |  |  |  |  |  |  |  |
| 14:1/22:1 |  |  |  |  |  |  |  |  |  |  |  |  |  |  |  |
| 14:1/22:2 |  |  |  |  |  |  |  |  |  |  |  |  |  |  |  |
| 16:0/16:4 |  |  |  |  |  |  |  |  |  |  |  |  |  |  |  |
| 16:0/18:1 |  |  |  |  |  |  |  |  |  |  |  |  |  |  |  |
| 16:0/18:2 |  |  |  |  |  |  |  |  |  |  |  |  |  |  |  |
| 16:0/18:3 |  |  |  |  |  |  |  |  |  |  |  |  |  |  |  |
| 16:0/20:4 |  |  |  |  |  |  |  |  |  |  |  |  |  |  |  |
| 16:0/22:1 |  |  |  |  |  |  |  |  |  |  |  |  |  |  |  |
| 16:0/22:4 |  |  |  |  |  |  |  |  |  |  |  |  |  |  |  |
| 16:0/24:1 |  |  |  |  |  |  |  |  |  |  |  |  |  |  |  |
| 16:0/24:2 |  |  |  |  |  |  |  |  |  |  |  |  |  |  |  |
| 16:0/24:3 |  |  |  |  |  |  |  |  |  |  |  |  |  |  |  |
| 16:0/24:4 |  |  |  |  |  |  |  |  |  |  |  |  |  |  |  |
| 16:1/20:0 |  |  |  |  |  |  |  |  |  |  |  |  |  |  |  |
| 16:1/20:1 |  |  |  |  |  |  |  |  |  |  |  |  |  |  |  |
| 16:1/24:4 |  |  |  |  |  |  |  |  |  |  |  |  |  |  |  |
| 16:2/24:4 |  |  |  |  |  |  |  |  |  |  |  |  |  |  |  |
| 16:3/20:1 |  |  |  |  |  |  |  |  |  |  |  |  |  |  |  |
| 16:3/22:1 |  |  |  |  |  |  |  |  |  |  |  |  |  |  |  |
| 16:3/24:2 |  |  |  |  |  |  |  |  |  |  |  |  |  |  |  |
| 16:3/24:3 |  |  |  |  |  |  |  |  |  |  |  |  |  |  |  |
| 16:3/24:4 |  |  |  |  |  |  |  |  |  |  |  |  |  |  |  |
| 16:4/18:3 |  |  |  |  |  |  |  |  |  |  |  |  |  |  |  |
| 16:4/22:1 |  |  |  |  |  |  |  |  |  |  |  |  |  |  |  |
| 16:4/24:1 |  |  |  |  |  |  |  |  |  |  |  |  |  |  |  |
| 16:4/24:4 |  |  |  |  |  |  |  |  |  |  |  |  |  |  |  |
| 18:0/22:1 |  |  |  |  |  |  |  |  |  |  |  |  |  |  |  |
| 18:1/20:1 |  |  |  |  |  |  |  |  |  |  |  |  |  |  |  |
| 18:1/22:1 |  |  |  |  |  |  |  |  |  |  |  |  |  |  |  |
| 18:1/22:4 |  |  |  |  |  |  |  |  |  |  |  |  |  |  |  |
| 18:1/24:2 |  |  |  |  |  |  |  |  |  |  |  |  |  |  |  |
| 18:2/22:1 |  |  |  |  |  |  |  |  |  |  |  |  |  |  |  |
| 18:2/22:3 |  |  |  |  |  |  |  |  |  |  |  |  |  |  |  |
| 18:2/22:4 |  |  |  |  |  |  |  |  |  |  |  |  |  |  |  |
| 18:2/24:2 |  |  |  |  |  |  |  |  |  |  |  |  |  |  |  |
| 18:2/24:4 |  |  |  |  |  |  |  |  |  |  |  |  |  |  |  |
| 18:3/20:1 |  |  |  |  |  |  |  |  |  |  |  |  |  |  |  |
| 18:3/22:1 |  |  |  |  |  |  |  |  |  |  |  |  |  |  |  |
| 18:3/22:2 |  |  |  |  |  |  |  |  |  |  |  |  |  |  |  |
| 18:3/22:3 |  |  |  |  |  |  |  |  |  |  |  |  |  |  |  |
| 18:3/22:4 |  |  |  |  |  |  |  |  |  |  |  |  |  |  |  |
| 18:3/24:2 |  |  |  |  |  |  |  |  |  |  |  |  |  |  |  |
| 18:3/24:3 |  |  |  |  |  |  |  |  |  |  |  |  |  |  |  |
| 18:3/24:4 |  |  |  |  |  |  |  |  |  |  |  |  |  |  |  |
| 18:4/20:0 |  |  |  |  |  |  |  |  |  |  |  |  |  |  |  |
| 18:4/22:0 |  |  |  |  |  |  |  |  |  |  |  |  |  |  |  |
| 18:4/24:4 |  |  |  |  |  |  |  |  |  |  |  |  |  |  |  |

*Fig. S2. Matrices showing the isoform profiles of PI and PIPs from* Desmodesmus quadricauda *in the samples used in the present study, through the cell cycle of* D. quadricauda, *using mass spectrometry. White indicates that the isoform was not found in any of the samples, green indicates that that isoform was found in all samples tested and blue that it was found in one sample. Collection points: +2 h, early part of G_1_; +4 h, mid-G_1_ (first CP); +6 h, end of G_1_ and pS; +8 h, S and second CP; +10 h, G_2_; +13 h, M; +15 h, G_3_, for others see* Figure 1*.*

|  | PC | | | | | | |  | PG | | | | | | |  | PE | | | | | | | |  |
| --- | --- | --- | --- | --- | --- | --- | --- | --- | --- | --- | --- | --- | --- | --- | --- | --- | --- | --- | --- | --- | --- | --- | --- | --- | --- |
|  | +2h | +4h | +6h | +8h | +10h | +13h | +15h |  | +2h | +4h | +6h | +8h | +10h | +13h | +15h |  | | +2h | +4h | +6h | +8h | +10h | +13h | +15h | |
| 14:0/20:4 |  |  |  |  |  |  |  |  |  |  |  |  |  |  |  |  | |  |  |  |  |  |  |  | |
| 14:2/20:1 |  |  |  |  |  |  |  |  |  |  |  |  |  |  |  |  | |  |  |  |  |  |  |  | |
| 14:2/20:3 |  |  |  |  |  |  |  |  |  |  |  |  |  |  |  |  | |  |  |  |  |  |  |  | |
| 16:0/16:0 |  |  |  |  |  |  |  |  |  |  |  |  |  |  |  |  | |  |  |  |  |  |  |  | |
| 16:0/16:1 |  |  |  |  |  |  |  |  |  |  |  |  |  |  |  |  | |  |  |  |  |  |  |  | |
| 16:0/16:2 |  |  |  |  |  |  |  |  |  |  |  |  |  |  |  |  | |  |  |  |  |  |  |  | |
| 16:0/16:3 |  |  |  |  |  |  |  |  |  |  |  |  |  |  |  |  | |  |  |  |  |  |  |  | |
| 16:0/16:4 |  |  |  |  |  |  |  |  |  |  |  |  |  |  |  |  | |  |  |  |  |  |  |  | |
| 16:0/18:0 |  |  |  |  |  |  |  |  |  |  |  |  |  |  |  |  | |  |  |  |  |  |  |  | |
| 16:0/18:1 |  |  |  |  |  |  |  |  |  |  |  |  |  |  |  |  | |  |  |  |  |  |  |  | |
| 16:0/18:2 |  |  |  |  |  |  |  |  |  |  |  |  |  |  |  |  | |  |  |  |  |  |  |  | |
| 16:0/18:3 |  |  |  |  |  |  |  |  |  |  |  |  |  |  |  |  | |  |  |  |  |  |  |  | |
| 16:0/18:4 |  |  |  |  |  |  |  |  |  |  |  |  |  |  |  |  | |  |  |  |  |  |  |  | |
| 16:0/20:3 |  |  |  |  |  |  |  |  |  |  |  |  |  |  |  |  | |  |  |  |  |  |  |  | |
| 16:0/20:4 |  |  |  |  |  |  |  |  |  |  |  |  |  |  |  |  | |  |  |  |  |  |  |  | |
| 16:0/22:0 |  |  |  |  |  |  |  |  |  |  |  |  |  |  |  |  | |  |  |  |  |  |  |  | |
| 16:0/22:3 |  |  |  |  |  |  |  |  |  |  |  |  |  |  |  |  | |  |  |  |  |  |  |  | |
| 16:0/22:4 |  |  |  |  |  |  |  |  |  |  |  |  |  |  |  |  | |  |  |  |  |  |  |  | |
| 16:0/24:0 |  |  |  |  |  |  |  |  |  |  |  |  |  |  |  |  | |  |  |  |  |  |  |  | |
| 16:0/24:1 |  |  |  |  |  |  |  |  |  |  |  |  |  |  |  |  | |  |  |  |  |  |  |  | |
| 16:0/24:2 |  |  |  |  |  |  |  |  |  |  |  |  |  |  |  |  | |  |  |  |  |  |  |  | |
| 16:0/24:3 |  |  |  |  |  |  |  |  |  |  |  |  |  |  |  |  | |  |  |  |  |  |  |  | |
| 16:1/18:1 |  |  |  |  |  |  |  |  |  |  |  |  |  |  |  |  | |  |  |  |  |  |  |  | |
| 16:1/18:2 |  |  |  |  |  |  |  |  |  |  |  |  |  |  |  |  | |  |  |  |  |  |  |  | |
| 16:1/18:3 |  |  |  |  |  |  |  |  |  |  |  |  |  |  |  |  | |  |  |  |  |  |  |  | |
| 16:1/18:4 |  |  |  |  |  |  |  |  |  |  |  |  |  |  |  |  | |  |  |  |  |  |  |  | |
| 16:1/22:0 |  |  |  |  |  |  |  |  |  |  |  |  |  |  |  |  | |  |  |  |  |  |  |  | |
| 16:1/24:0 |  |  |  |  |  |  |  |  |  |  |  |  |  |  |  |  | |  |  |  |  |  |  |  | |
| 16:2/18:1 |  |  |  |  |  |  |  |  |  |  |  |  |  |  |  |  | |  |  |  |  |  |  |  | |
| 16:2/18:2 |  |  |  |  |  |  |  |  |  |  |  |  |  |  |  |  | |  |  |  |  |  |  |  | |
| 16:2/18:3 |  |  |  |  |  |  |  |  |  |  |  |  |  |  |  |  | |  |  |  |  |  |  |  | |
| 16:3/16:3 |  |  |  |  |  |  |  |  |  |  |  |  |  |  |  |  | |  |  |  |  |  |  |  | |
| 16:3/16:4 |  |  |  |  |  |  |  |  |  |  |  |  |  |  |  |  | |  |  |  |  |  |  |  | |
| 16:3/18:0 |  |  |  |  |  |  |  |  |  |  |  |  |  |  |  |  | |  |  |  |  |  |  |  | |
| 16:3/18:1 |  |  |  |  |  |  |  |  |  |  |  |  |  |  |  |  | |  |  |  |  |  |  |  | |
| 16:3/18:2 |  |  |  |  |  |  |  |  |  |  |  |  |  |  |  |  | |  |  |  |  |  |  |  | |
| 16:3/18:3 |  |  |  |  |  |  |  |  |  |  |  |  |  |  |  |  | |  |  |  |  |  |  |  | |
| 16:3/18:4 |  |  |  |  |  |  |  |  |  |  |  |  |  |  |  |  | |  |  |  |  |  |  |  | |
| 16:4/16:4 |  |  |  |  |  |  |  |  |  |  |  |  |  |  |  |  | |  |  |  |  |  |  |  | |
| 16:4/18:1 |  |  |  |  |  |  |  |  |  |  |  |  |  |  |  |  | |  |  |  |  |  |  |  | |
| 16:4/18:2 |  |  |  |  |  |  |  |  |  |  |  |  |  |  |  |  | |  |  |  |  |  |  |  | |
| 16:4/18:3 |  |  |  |  |  |  |  |  |  |  |  |  |  |  |  |  | |  |  |  |  |  |  |  | |
| 16:4/18:4 |  |  |  |  |  |  |  |  |  |  |  |  |  |  |  |  | |  |  |  |  |  |  |  | |
| 16:4/20:0 |  |  |  |  |  |  |  |  |  |  |  |  |  |  |  |  | |  |  |  |  |  |  |  | |
| 16:4/20:2 |  |  |  |  |  |  |  |  |  |  |  |  |  |  |  |  | |  |  |  |  |  |  |  | |
| 16:4/20:4 |  |  |  |  |  |  |  |  |  |  |  |  |  |  |  |  | |  |  |  |  |  |  |  | |
| 16:4/24:1 |  |  |  |  |  |  |  |  |  |  |  |  |  |  |  |  | |  |  |  |  |  |  |  | |
| 16:4/24:3 |  |  |  |  |  |  |  |  |  |  |  |  |  |  |  |  | |  |  |  |  |  |  |  | |
| 16:4/24:4 |  |  |  |  |  |  |  |  |  |  |  |  |  |  |  |  | |  |  |  |  |  |  |  | |

|  | PC | | | | | | |  | PG | | | | | | |  | PE | | | | | | |
| --- | --- | --- | --- | --- | --- | --- | --- | --- | --- | --- | --- | --- | --- | --- | --- | --- | --- | --- | --- | --- | --- | --- | --- |
|  | +2h | +4h | +6h | +8h | +10h | +13h | +15h |  | +2h | +4h | +6h | +8h | +10h | +13h | +15h |  | +2h | +4h | +6h | +8h | +10h | +13h | +15h |
| 18:1/18:1 |  |  |  |  |  |  |  |  |  |  |  |  |  |  |  |  |  |  |  |  |  |  |  |
| 18:1/18:2 |  |  |  |  |  |  |  |  |  |  |  |  |  |  |  |  |  |  |  |  |  |  |  |
| 18:1/18:3 |  |  |  |  |  |  |  |  |  |  |  |  |  |  |  |  |  |  |  |  |  |  |  |
| 18:1/18:4 |  |  |  |  |  |  |  |  |  |  |  |  |  |  |  |  |  |  |  |  |  |  |  |
| 18:1/24:0 |  |  |  |  |  |  |  |  |  |  |  |  |  |  |  |  |  |  |  |  |  |  |  |
| 18:1/24:1 |  |  |  |  |  |  |  |  |  |  |  |  |  |  |  |  |  |  |  |  |  |  |  |
| 18:1/24:2 |  |  |  |  |  |  |  |  |  |  |  |  |  |  |  |  |  |  |  |  |  |  |  |
| 18:1/24:3 |  |  |  |  |  |  |  |  |  |  |  |  |  |  |  |  |  |  |  |  |  |  |  |
| 18:1/24:4 |  |  |  |  |  |  |  |  |  |  |  |  |  |  |  |  |  |  |  |  |  |  |  |
| 18:2/18:2 |  |  |  |  |  |  |  |  |  |  |  |  |  |  |  |  |  |  |  |  |  |  |  |
| 18:2/18:3 |  |  |  |  |  |  |  |  |  |  |  |  |  |  |  |  |  |  |  |  |  |  |  |
| 18:2/18:4 |  |  |  |  |  |  |  |  |  |  |  |  |  |  |  |  |  |  |  |  |  |  |  |
| 18:2/20:3 |  |  |  |  |  |  |  |  |  |  |  |  |  |  |  |  |  |  |  |  |  |  |  |
| 18:2/22:3 |  |  |  |  |  |  |  |  |  |  |  |  |  |  |  |  |  |  |  |  |  |  |  |
| 18:2/22:4 |  |  |  |  |  |  |  |  |  |  |  |  |  |  |  |  |  |  |  |  |  |  |  |
| 18:2/24:1 |  |  |  |  |  |  |  |  |  |  |  |  |  |  |  |  |  |  |  |  |  |  |  |
| 18:3/18:3 |  |  |  |  |  |  |  |  |  |  |  |  |  |  |  |  |  |  |  |  |  |  |  |
| 18:3/18:4 |  |  |  |  |  |  |  |  |  |  |  |  |  |  |  |  |  |  |  |  |  |  |  |
| 18:3/20:0 |  |  |  |  |  |  |  |  |  |  |  |  |  |  |  |  |  |  |  |  |  |  |  |
| 18:3/20:1 |  |  |  |  |  |  |  |  |  |  |  |  |  |  |  |  |  |  |  |  |  |  |  |
| 18:3/20:4 |  |  |  |  |  |  |  |  |  |  |  |  |  |  |  |  |  |  |  |  |  |  |  |
| 18:3/22:0 |  |  |  |  |  |  |  |  |  |  |  |  |  |  |  |  |  |  |  |  |  |  |  |
| 18:3/22:1 |  |  |  |  |  |  |  |  |  |  |  |  |  |  |  |  |  |  |  |  |  |  |  |
| 18:3/22:3 |  |  |  |  |  |  |  |  |  |  |  |  |  |  |  |  |  |  |  |  |  |  |  |
| 18:3/22:3 |  |  |  |  |  |  |  |  |  |  |  |  |  |  |  |  |  |  |  |  |  |  |  |
| 18:3/22:4 |  |  |  |  |  |  |  |  |  |  |  |  |  |  |  |  |  |  |  |  |  |  |  |
| 18:3/24:0 |  |  |  |  |  |  |  |  |  |  |  |  |  |  |  |  |  |  |  |  |  |  |  |
| 18:3/24:1 |  |  |  |  |  |  |  |  |  |  |  |  |  |  |  |  |  |  |  |  |  |  |  |
| 18:3/24:2 |  |  |  |  |  |  |  |  |  |  |  |  |  |  |  |  |  |  |  |  |  |  |  |
| 18:3/24:3 |  |  |  |  |  |  |  |  |  |  |  |  |  |  |  |  |  |  |  |  |  |  |  |
| 18:3/24:4 |  |  |  |  |  |  |  |  |  |  |  |  |  |  |  |  |  |  |  |  |  |  |  |
| 18:4/18:4 |  |  |  |  |  |  |  |  |  |  |  |  |  |  |  |  |  |  |  |  |  |  |  |
| 18:4/22:4 |  |  |  |  |  |  |  |  |  |  |  |  |  |  |  |  |  |  |  |  |  |  |  |

*Fig. S3. Matrix showing the frequency of the detection of isoforms of PC, PG and PE in the samples used in the present study, through the cell cycle of* D. quadricauda, *using mass spectrometry. White indicates that the isoform was not found in any of the samples, green indicates that the isoform was found in all samples tested and blue that it was found in one sample. Collection points: +2 h, early part of G_1_; +4 h, mid-G_1_ (first CP); +6 h, end of G_1_ and pS; +8 h, S and second CP; +10 h, G_2_; +13 h, M; +15 h, G_3_, for others see* Figure 1*.*

*
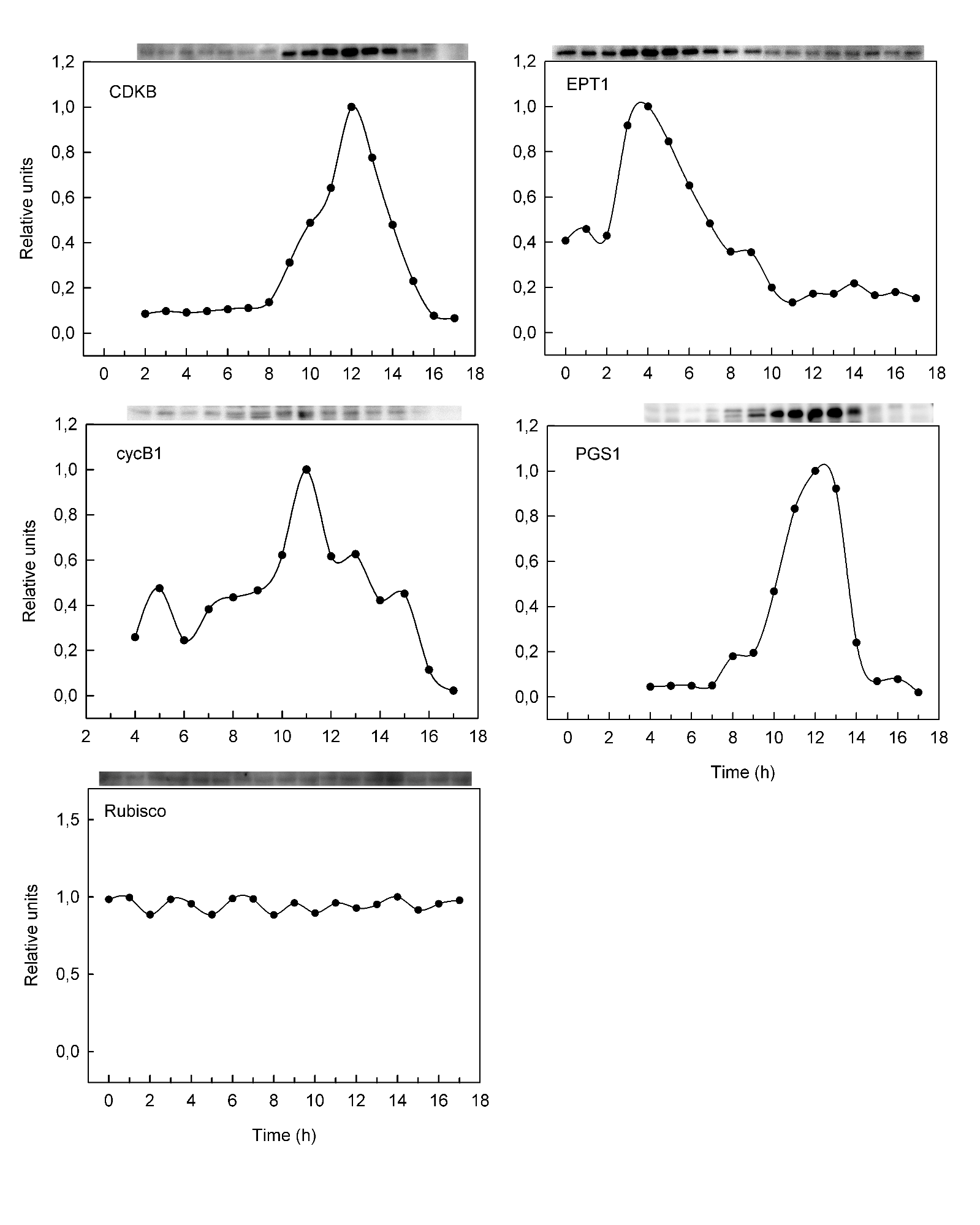
*

*Fig. S4. Western immunoblots and accompanying plots of relative abundance of ethanolamine phosphate transferase (EPT1) and phosphatidylglycerol synthase (PGS1) through a typical cell cycle (as demonstrated by the abundance of CDKB and cycB1) and against a steady rate of protein synthesis (as demonstrated by the abundance of constitutive protein RuBISCo). X Data = time (h); Y Data = relative abundance (a.u.).*

**Supplementary Tables**

|  |  | Collection point | | | | | | | | | | | | | |
| --- | --- | --- | --- | --- | --- | --- | --- | --- | --- | --- | --- | --- | --- | --- | --- |
|  |  | +2 h | | +4h | | +6 h | | +8 h | | +10 h | | +13 h | | +15 h | |
| ID | ^31^P NMR Shift | **Mean (%)** | **+/-** | **Mean (%)** | **+/-** | **Mean (%)** | **+/-** | **Mean (%)** | **+/-** | **Mean (%)** | **+/-** | **Mean (%)** | **+/-** | **Mean (%)** | **+/-** |
| PA | 5·15 | 0·6 | 0·6 | 0·3 | 0·1 | 0·6 | 0·5 | 0·8 | 0·6 | 0·5 | 0·3 | 1·1 | 0·6 | 4·1 | 1·2 |
| PG | 1·22 | 16·0 | 2·9 | 16·8 | 5·0 | 19·4 | 4·9 | 28·7 | 6·3 | 28·4 | 7·4 | 24·6 | 5·5 | 23·7 |  |
| PI | 1·07 | 5·8 | 1·5 | 4·3 | 1·8 | 5·9 | 0·9 | 5·9 | 1·2 | 6·0 | 1·5 | 5·3 | 1·4 | 5·9 | 0·8 |
| *lyso*-PE | 0·93 | 1·5 | 1·1 | 1·8 | 0·2 | 1·3 | 1·1 | 1·2 | 0·9 | 0·9 | 0·8 | 0·3 | 0·3 | 0·8 | 0·7 |
| U | 0·80 | 2·3 | 1·6 | 0·6 | 0·4 | 0·8 | 0·5 | 1·1 | 1·0 | 0·7 | 0·7 | 1·4 | 1·0 | 1·6 | 1·0 |
| CL | 0·78 | 1·2 | 1·3 | 0·8 | 0·7 | 0·4 | 0·1 | 0·4 | 0·3 | 0·6 | 0·6 | 1·5 | 2·0 | 0·9 | 0·5 |
| U | 0·68 | 2·8 | 1·5 | 3·1 | 1·2 | 2·4 | 2·0 | 2·8 | 1·6 | 2·7 | 2·1 | 2·4 | 1·8 | 1·8 | 1·3 |
| PE-plas. | 0·59 | 0·9 | 0·6 | 1·3 | 1·3 | 1·2 | 0·5 | 1·4 | 1·9 | 0·5 | 0·5 | 1·7 | 2·0 | 1·0 | 0·4 |
| PS | 0·51 | 1·1 | 1·1 | 2·4 | 2·4 | ND | - | 0·6 | 0·5 | 3·6 | 0·3 | 1·0 | 0·8 | 0·8 | 0·5 |
| *lyso*-PC | 0·47 | 1·1 | 0·8 | 4·7 | 5·0 | 0·5 | 0·5 | 2·3 | 3·2 | ND | - | ND | - | 1·3 | 1·7 |
| PC-plas. | 0·22 | 1·0 | 1·2 | 1·0 | 0·2 | 2·6 | 2·0 | 1·4 | 2·6 | 1·7 | 2·3 | 1·7 | 0·7 | 4·4 | 0·4 |
| PE | Var. | 26·7 | 5·1 | 30·6 | 2·8 | 36·9 | 2·3 | 27·8 | 2·1 | 28·0 | 5·3 | 26·5 | 4·5 | 25·9 | 3·1 |
| PC | 0·00 | 38·9 | 1·7 | 32·2 | 3·6 | 26·7 | 4·1 | 24·2 | 1·8 | 26·0 | 3·0 | 26·6 | 1·8 | 27·7 | 1·1 |

*Table S1. The lipid head group profile of* D. quadricauda *determined using ^31^P NMR. ^31^P assignments made using literature references [*[*30-32*](#_ENREF_30)*]. CL, cardiolipin; PA, phosphatidic acid; PC, phosphatidylcholine; PE, phosphatidylethanolamine; PG, phosphatidylglycerol; PI, phosphatidylinositol; PS, phosphatidylserine; U, unidentified. Collection points: +2 h, early part of G_1_; +4 h, mid-G_1_ (first CP); +6 h, end of G_1_ and pS; +8 h, S and second CP; +10 h, G_2_; +13 h, M; +15 h, G_3_, for others see* Figure 1*.*

|  | Collection point | | | | | | |
| --- | --- | --- | --- | --- | --- | --- | --- |
|  | +2 h | +4h | +6 h | +8 h | +10 h | +13 h | +15 h |
| PA(12:0/18:3) |  |  |  |  |  |  |  |
| PA(12:2/18:0) |  |  |  |  |  |  |  |
| PA(12:2/22:4) |  |  |  |  |  |  |  |
| PA(16:1/18:3) |  |  |  |  |  |  |  |
| PA(16:4/24:4) |  |  |  |  |  |  |  |
| PA(18:2/24:4) |  |  |  |  |  |  |  |
| PA(18:3/20:1) |  |  |  |  |  |  |  |
| PA(18:3/20:2) |  |  |  |  |  |  |  |
| PA(18:3/24:2) |  |  |  |  |  |  |  |
| PA(18:3/24:3) |  |  |  |  |  |  |  |
| PA(18:3/24:4) |  |  |  |  |  |  |  |
| PA(18:4/24:4) |  |  |  |  |  |  |  |

*Table S2. The Isoform profile of phosphatidic acids from* D. quadricauda *determined using MS/MS. White indicates not found, blue indicates found in one or more samples, green indicates found in all samples tested. Collection points: +2 h, early part of G_1_; +4 h, mid-G_1_ (first CP); +6 h, end of G_1_ and pS; +8 h, S and second CP; +10 h, G_2_; +13 h, M; +15 h, G_3_, for others see* Figure 1*.*

|  | Collection point | | | | | | |
| --- | --- | --- | --- | --- | --- | --- | --- |
|  | +2 h | +4h | +6 h | +8 h | +10 h | +13 h | +15 h |
| PS(14:1/18:3) |  |  |  |  |  |  |  |
| PS(14:1/18:4) |  |  |  |  |  |  |  |
| PS(16:0/16:0) |  |  |  |  |  |  |  |
| PS(16:0/16:3) |  |  |  |  |  |  |  |
| PS(16:0/16:4) |  |  |  |  |  |  |  |
| PS(16:0/18:0) |  |  |  |  |  |  |  |
| PS(16:0/18:1) |  |  |  |  |  |  |  |
| PS(16:0/18:2) |  |  |  |  |  |  |  |
| PS(16:0/18:3) |  |  |  |  |  |  |  |
| PS(16:0/22:4) |  |  |  |  |  |  |  |
| PS(16:0/24:4) |  |  |  |  |  |  |  |
| PS(16:1/16:4) |  |  |  |  |  |  |  |
| PS(16:1/18:1) |  |  |  |  |  |  |  |
| PS(16:1/18:2) |  |  |  |  |  |  |  |
| PS(16:1/18:3) |  |  |  |  |  |  |  |
| PS(16:2/16:4) |  |  |  |  |  |  |  |
| PS(16:2/18:1) |  |  |  |  |  |  |  |
| PS(16:2/18:2) |  |  |  |  |  |  |  |
| PS(16:3/16:4) |  |  |  |  |  |  |  |
| PS(16:3/18:1) |  |  |  |  |  |  |  |
| PS(16:3/18:2) |  |  |  |  |  |  |  |
| PS(16:3/18:3) |  |  |  |  |  |  |  |
| PS(16:3/24:4) |  |  |  |  |  |  |  |
| PS(16:4/16:4) |  |  |  |  |  |  |  |
| PS(16:4/18:1) |  |  |  |  |  |  |  |
| PS(16:4/18:2) |  |  |  |  |  |  |  |
| PS(16:4/18:3) |  |  |  |  |  |  |  |
| PS(16:4/18:4) |  |  |  |  |  |  |  |
| PS(18:1/18:2) |  |  |  |  |  |  |  |
| PS(18:1/18:3) |  |  |  |  |  |  |  |
| PS(18:1/18:4) |  |  |  |  |  |  |  |
| PS(18:2/18:3) |  |  |  |  |  |  |  |
| PS(18:3/18:3) |  |  |  |  |  |  |  |
| PS(18:3/18:4) |  |  |  |  |  |  |  |
| PS(18:3/22:4) |  |  |  |  |  |  |  |

*Table S3. The Isoform profile of phosphatidylserines from* D. quadricauda *determined using MS/MS. White indicates not found, blue indicates found in one sample, green indicates found in both samples tested. Collection points: +2 h, early part of G_1_; +4 h, mid-G_1_ (first CP); +6 h, end of G_1_ and pS; +8 h, S and second CP; +10 h, G_2_; +13 h, M; +15 h, G_3_, for others see* Figure 1*.*

|  |  | Collection point | | | | | | |
| --- | --- | --- | --- | --- | --- | --- | --- | --- |
| Lipid annotation | *m/z* | +2 h | +4h | +6 h | +8 h | +10 h | +13 h | +15 h |
| Cer(t34:0)_[M+H]+ / Cer(d34:0 (2-OH)) [M+H]+ | 556·5305 | 0·09 | 0·23 | 0·34 | 0·19 | 0·19 | 0·30 | 0·29 |
| DGDG(36:8)_[M+K]+ | 971·5129 | 0·76 | 1·02 | 0·85 | 0·59 | 0·55 | 0·78 | 0·53 |
| DGDG(36:8)_[M+NH4]+ | 950·5835 | 2·86 | 4·81 | 4·81 | 4·86 | 5·75 | 4·51 | 3·61 |
| MGDG(34:1)_[M+G]+ | 851·6002 | 0·11 | 2·34 | 0·69 | 0·89 | 1·23 | 1·08 | 1·47 |
| MGDG(34:1)_[M+H]+ | 757·5830 | 73·35 | 62·13 | 63·78 | 54·15 | 67·31 | 78·78 | 73·46 |
| MGDG(34:2)_[M+H]+ | 755·5673 | 110·15 | 63·89 | 56·57 | 31·35 | 42·60 | 63·10 | 72·65 |
| MGDG(34:6)_[M+NH4]+ | 764·5307 | 57·75 | 64·32 | 73·96 | 85·58 | 91·82 | 83·34 | 69·68 |
| MGDG(34:6)_[M+Na]+/ MGDG(36:9) [M+H]+ | 769·4867 | 60·30 | 55·19 | 69·52 | 51·02 | 44·76 | 53·78 | 18·24 |
| MGDG(36:10)_[M+Na]+ | 789·4554 | 1·96 | 1·49 | 1·27 | 1·46 | 1·47 | 1·72 | 0·71 |
| MGDG(36:5)_[M+G]+ | 871·5689 | 158·14 | 152·22 | 150·22 | 221·00 | 239·27 | 210·66 | 176·93 |
| MGDG(36:6)_[M+G]+ | 869·5532 |  | 0·21 |  | 38·84 | 28·40 | 10·95 | 14·82 |
| MGDG(36:6)_[M+NH4]+ | 792·5620 | 3·87 | 5·81 | 5·71 | 7·72 | 5·89 | 2·23 | 2·99 |
| MGDG(36:6_[M+Na]+/ MGDG(38:9) [M+H]+ | 797·5180 | 10·61 | 10·27 | 14·68 | 10·15 | 6·37 | 5·05 | 2·26 |
| MGDG(36:8)_[M+G]+ | 865·5219 | 2·02 | 1·55 | 1·63 | 6·48 | 4·93 | 3·08 | 3·53 |
| MGDG(36:8)_[M+Na]+/ MGDG(38:11) [M+H]+ | 793·4867 | 5·80 | 4·41 | 4·29 | 5·26 | 4·18 | 3·94 | 1·69 |
| MGDG(36:9)_[M+Na]+ | 791·4711 | 1·81 | 2·71 | 2·46 | 1·48 | 1·14 | 1·14 | 1·13 |
| MGDG(38:8)_[M+G]+ | 893·5532 | 36·79 | 32·34 | 32·35 | 41·87 | 45·20 | 50·84 | 23·92 |
| MGDG(38:9)_[M+G]+ | 891·5376 | 3·49 | 2·75 | 2·22 | 15·08 | 11·62 | 10·07 | 5·64 |
| MGDG-O(16:03)_[M+K]+ | 799·4394 | 7·48 | 5·66 | 6·17 | 6·20 | 5·43 | 4·62 | 1·64 |
| MGDG-O(16:03)_[M+G]+ | 855·5012 | 0·57 | 0·10 | 0·29 | 0·14 |  | 0·55 | 0·47 |
| SQDG(32:0)_[M+NH4]+ | 812·5552 | 15·37 | 15·93 | 16·59 | 13·14 | 14·10 | 14·17 | 9·55 |
| SQDG(32:0)_[M+Na]+/ SQDG(34:2) [M+H]+ | 817·5112 | 14·18 | 12·62 | 16·73 | 10·12 | 9·36 | 10·23 | 5·51 |
| SQDG(32:1)_[M+H]+ | 793·5136 | 4·46 | 3·67 | 3·45 | 4·57 | 3·64 | 2·96 | 1·52 |
| SQDG(32:1)_[M+Na]+/ SQDG(34:3) [M+H]+ | 815·4956 |  |  |  |  |  |  | 0·05 |

*Table S4. Signal intensities for high resolution* m/z *values consistent with isoforms of galactosyl-diglycerides and one phosphatidylinositol-ceramide (n = 2). Signals were assigned according to high resolution masses with a deviation of <12 ppm from the calculated monoisotopic mass and checked with a visual inspection of a subset of the original chromatograms. CE, Campestryl ester; G, guanidine; MGDG, mono-galactosyl diglyceride; MGDG-O, mono-galactosyl diglyceride oxide; PI-Cer, phosphatidylinositol ceramide; SQDG, sulfoquinovosyl diglyceride; TG, triglyceride. Collection points: +2 h, early part of G_1_; +4 h, mid-G_1_ (first CP); +6 h, end of G_1_ and pS; +8 h, S and second CP; +10 h, G_2_; +13 h, M; +15 h, G_3_, for others see* Figure 1*.*

|  |  | Collection point | | | | | | |
| --- | --- | --- | --- | --- | --- | --- | --- | --- |
| Triglyceride annotation | *m/z* | +2 h | +4h | +6 h | +8 h | +10 h | +13 h | +15 h |
| TG(30:00)_[M+Na]^+^ | 577·4439 | 5·64 | 3·92 | 3·15 | 7·22 | 10·76 | 9·92 | 12·32 |
| TG(30:00)_[M+NH_4_]^+^ | 572·4885 | 1·75 | 3·76 | 6·55 | 7·18 | 4·86 | 3·15 | 3·21 |
| TG(34:00)_[M+NH_4_]^+^ | 628·5515 | 0·20 | 0·24 | 0·14 | 0·26 | 0·24 | 0·22 | 0·26 |
| TG(34:01)_[M+NH_4_]^+^ | 626·5359 | 0·12 | 0·07 | 0·04 | 0·13 | 0·16 | 0·13 | 0·12 |
| TG(35:00)_[M+NH_4_]^+^ | 642·5672 | 0·29 | 0·30 | 0·54 | 0·47 | 0·30 | 0·54 | 0·54 |
| TG(35:01)_[M+NH_4_]^+^ | 640·5515 | 0·29 | 0·32 | 0·43 | 0·46 | 0·28 |  | 0·45 |
| TG(36:02)_[M+NH_4_]^+^ | 652·5515 | 0·07 | 0·05 | 0·05 | 0·18 | 0·07 | 0·05 | 0·14 |
| TG(36:03)_[M+NH_4_]^+^ | 650·5359 |  |  |  | 0·04 |  | 0·05 | 0·03 |
| TG(37:00)_[M+Na]^+^ | 675·5534 | 0·63 | 0·35 | 0·07 |  | 0·11 | 0·17 | 0·79 |
| TG(37:01)_[M+NH_4_]^+^ | 668·5828 | 0·27 | 0·20 | 0·20 | 0·14 | 0·14 | 0·29 | 0·25 |
| TG(38:03)_[M+NH_4_]^+^ | 678·5672 | 0·58 | 0·39 | 0·33 | 0·08 |  |  | 0·28 |
| TG(38:04)_[M+NH_4_]^+^ | 676·5515 | 1·42 | 0·87 | 0·47 | 0·31 | 0·36 | 0·40 | 1·40 |
| TG(40:01)_[M+Na]^+^ | 715·5847 | 0·39 | 0·47 | 0·28 | 0·64 | 0·74 | 0·52 | 0·51 |
| TG(40:02)_[M+Na]^+^ | 713·5691 | 0·38 | 0·37 | 0·21 | 0·43 | 0·71 | 0·60 | 0·99 |
| TG(40:02)_[M+NH_4_]^+^ | 708·6137 | 4·72 | 4·65 | 3·73 | 4·98 | 4·69 | 4·19 |  |
| TG(40:04)_[M+NH_4_]^+^ | 704·5828 | 48·79 | 49·22 | 40·54 | 28·16 |  | 33·77 | 51·10 |
| TG(40:05)_[M+NH_4_]^+^ | 702·5672 |  | 1·52 | 0·67 | 0·80 | 0·03 |  |  |
| TG(40:06)_[M+NH_4_]^+^ | 700·5515 | 0·49 | 0·37 | 0·57 |  | 0·63 | 0·59 |  |
| TG(41:00)_[M+Na]^+^ | 731·6160 | 6·13 | 3·35 | 1·95 | 3·64 | 7·70 | 10·25 | 16·76 |
| TG(41:00)_[M+NH_4_]^+^ | 726·6606 | 43·15 | 31·67 | 24·21 | 19·28 | 28·61 | 33·59 | 34·90 |
| TG(41:01)_[M+Na]^+^ | 729·6004 | 11·83 | 6·70 | 5·16 | 5·16 | 9·60 | 12·29 | 17·78 |
| TG(41:02)_[M+Na]^+^ | 727·5847 | 29·75 | 18·52 | 12·87 | 10·05 | 16·63 | 21·75 | 24·94 |
| TG(41:02)_[M+NH_4_]^+^ | 722·6293 | 3·47 | 2·61 | 1·87 | 0·96 | 1·86 | 1·83 | 3·04 |
| TG(41:03)_[M+NH_4_]^+^ | 720·6141 | 3·06 | 2·58 | 1·91 | 1·38 | 2·23 | 2·02 | 3·39 |
| TG(42:01)_[M+Na]^+^ | 743·6160 | 1·02 | 1·07 | 1·01 | 2·19 | 2·68 | 2·11 | 3·47 |
| TG(42:02)_[M+Na]^+^ | 741·6004 | 2·48 | 2·27 | 3·00 | 4·46 | 4·13 | 3·03 | 2·70 |
| TG(42:03)_[M+Na]^+^ | 739·5847 | 4·37 | 4·08 | 4·39 | 7·37 | 8·59 |  | 8·59 |
| TG(42:03)_[M+NH_4_]^+^ | 734·6298 | 110·24 | 108·80 | 98·60 |  | 145·05 | 104·14 | 139·80 |
| TG(42:04)_[M+NH_4_]^+^ | 732·6137 | 107·47 | 113·34 | 116·64 | 125·71 |  | 102·35 |  |
| TG(42:05)_[M+NH_4_]^+^ | 730·5985 | 14·04 | 7·24 | 4·54 | 6·96 | 15·84 | 21·87 | 41·41 |
| TG(42:06)_[M+NH_4_]^+^ | 728·5828 | 29·23 | 16·62 | 12·02 | 11·27 | 20·86 | 27·79 | 37·83 |
| TG(43:02)_[M+Na]^+^ | 755·6160 | 47·85 | 26·47 | 22·30 | 17·75 | 21·63 | 26·09 | 37·00 |
| TG(43:04)_[M+NH_4_]^+^ | 746·6293 | 3·56 | 3·42 | 2·65 | 3·12 | 3·93 | 3·60 | 4·92 |
| TG(44:01)_[M+Na]^+^ | 771·6473 | 9·31 | 8·01 | 8·26 | 11·95 | 9·69 | 9·94 | 4·97 |
| TG(44:01)_[M+NH_4_]^+^ | 766·6919 | 11·34 | 10·26 | 9·15 | 14·94 | 18·00 | 14·34 | 14·19 |
| TG(44:02)_[M+Na]^+^ | 769·6317 | 25·86 | 19·99 | 22·86 | 22·63 | 18·23 | 18·95 | 8·36 |
| TG(44:03)_[M+Na]^+^ | 767·6160 | 33·53 | 28·23 | 37·64 | 35·71 | 40·19 | 31·10 | 13·70 |
| TG(44:05)_[M+NH_4_]^+^ | 758·6293 | 23·63 | 18·86 | 15·01 | 40·70 | 55·41 | 56·41 | 73·10 |
| TG(44:06)_[M+NH_4_]^+^ | 756·6141 | 74·42 | 53·90 | 53·06 | 55·86 | 62·84 | 64·63 | 74·92 |
| TG(45:00)_[M+NH_4_]^+^ | 782·7232 | 17·78 | 25·96 | 28·15 | 44·27 | 53·95 | 44·77 | 55·18 |
| TG(45:03)_[M+Na]^+^ | 781·6317 | 15·84 | 17·49 | 18·45 | 26·91 | 30·20 | 21·86 | 27·05 |
| TG(45:05)_[M+NH_4_]^+^ | 772·6450 | 5·73 | 3·84 | 3·52 | 5·78 | 5·38 | 6·00 | 3·91 |
| TG(45:06)_[M+NH_4_]^+^ | 770·6298 | 12·51 | 8·82 | 9·98 | 11·31 | 10·34 | 9·65 | 5·82 |
| TG(46:00)_[M+NH_4_]^+^ | 796·7389 | 2·04 | 1·62 | 2·29 | 2·05 | 1·11 | 0·81 | 0·62 |
| TG(46:02)_[M+Na]^+^ | 797·6630 | 4·58 | 3·70 | 5·03 | 4·55 | 2·56 | 1·82 | 0·82 |
| TG(46:03)_[M+Na]^+^ | 795·6473 | 0·84 | 0·63 | 0·74 | 0·62 | 0·52 | 0·28 | 0·44 |
| TG(46:06)_[M+NH_4_]^+^ | 784·6450 | 5·11 | 11·58 | 6·66 | 26·69 | 49·07 | 43·21 | 64·22 |
| TG(46:08)_[M+NH_4_]^+^ | 780·6141 | 25·14 | 30·17 | 28·29 | 44·64 | 53·16 | 36·45 | 45·80 |
| TG(47:00)_[M+NH_4_]^+^ | 810·7545 | 0·26 | 0·27 | 0·17 | 0·64 | 0·52 | 0·58 | 1·06 |
| TG(48:00)_[M+NH_4_]^+^ | 824·7702 | 0·26 | 0·59 | 0·21 | 0·25 | 0·18 | 0·09 | 0·40 |
| TG(48:02)_[M+NH_4_]^+^ | 820·7389 |  | 0·43 | 0·19 | 0·66 | 0·68 | 0·58 | 1·27 |
| TG(48:06)_[M+NH_4_]^+^ | 812·6763 | 5·92 | 4·63 | 4·95 | 4·74 | 4·68 | 4·06 | 3·55 |
| TG(49:00)_[M+Na]^+^ | 843·7412 | 0·33 | 0·43 | 1·02 | 0·49 | 1·05 | 2·73 | 1·25 |
| TG(49:03)_[M+Na]^+^ | 837·6943 | 1·39 | 1·24 | 0·97 | 3·32 | 3·10 | 3·80 | 8·72 |
| TG(49:07)_[M+Na]^+^ | 829·6317 | 1·07 | 0·54 |  | 0·02 | 0·15 | 0·06 | 0·46 |
| TG(49:08)_[M+NH_4_]^+^ | 822·6606 | 0·36 |  |  |  | 0·13 |  | 0·41 |
| TG(50:01)_[M+NH_4_]^+^ | 850·7858 | 0·19 | 0·15 | 0·89 | 3·59 |  | 0·09 | 0·34 |
| TG(50:03)_[M+NH_4_]^+^ | 846·7545 |  | 4·34 |  | 3·27 | 0·20 | 0·27 |  |
| TG(50:07)_[M+NH_4_]^+^ | 838·6919 | 1·67 | 1·22 | 0·98 | 2·26 | 3·28 | 2·69 | 4·35 |
| TG(50:10)_[M+NH_4_]^+^ | 832·6454 | 3·89 | 4·30 | 4·34 | 11·08 | 19·83 | 18·17 | 33·69 |
| TG(50:11)_[M+NH_4_]^+^ | 830·6298 | 4·08 | 4·13 | 3·81 | 11·13 | 22·21 | 19·98 | 29·81 |
| TG(51:00)_[M+Na]^+^ | 871·7725 | 16·72 | 20·04 | 3·47 | 28·34 | 29·59 | 12·54 | 21·44 |
| TG(51:01)_[M+Na]^+^ | 869·7569 | 0·33 | 0·96 |  | 3·67 | 4·77 |  |  |
| TG(51:01)_[M+NH_4_]^+^ | 864·8015 | 0·36 | 0·13 | 0·26 | 0·30 | 0·24 | 0·21 | 0·35 |
| TG(51:02)_[M+NH_4_]^+^ | 862·7858 | 0·13 | 0·27 | 0·61 | 0·16 | 0·25 | 0·25 | 0·34 |
| TG(51:06)_[M+Na]^+^ | 859·6786 | 0·58 | 1·44 | 1·11 | 1·85 | 1·87 | 1·67 | 1·68 |
| TG(51:07)_[M+Na]^+^ | 857·6630 | 0·62 | 0·08 |  |  | 0·21 | 0·15 | 0·20 |
| TG(52:02)_[M+NH_4_]^+^ | 876·8015 | 1·19 | 0·18 | 2·73 | 8·90 |  | 0·34 |  |
| TG(52:04)_[M+Na]^+^ | 877·7256 | 0·97 | 3·57 |  | 2·49 |  | 0·52 |  |
| TG(52:10)_[M+NH_4_]^+^ | 860·6763 | 1·38 | 1·70 | 1·75 | 1·86 | 2·05 | 1·87 | 2·06 |
| TG(52:11)_[M+NH_4_]^+^ | 858·6611 | 0·91 | 0·79 | 0·71 | 1·18 | 1·51 | 1·23 | 1·97 |
| TG(53:00)_[M+NH_4_]^+^ | 894·8484 | 5·54 | 5·68 | 3·55 | 8·81 | 7·81 | 7·81 | 3·87 |
| TG(53:02)_[M+NH_4_]^+^ | 890·8171 | 0·78 | 0·32 | 0·23 | 3·18 | 2·60 | 1·90 | 1·51 |
| TG(53:05)_[M+Na]^+^ | 889·7256 | 1·42 | 0·75 | 0·52 | 2·52 | 1·84 | 1·15 |  |
| TG(53:06)_[M+NH_4_]^+^ | 882·7545 | 0·21 | 0·08 | 0·49 | 1·19 | 0·15 |  | 0·19 |
| TG(53:07)_[M+Na]^+^ | 885·6943 | 2·00 | 0·16 | 0·26 | 8·89 | 20·82 | 13·04 |  |
| TG(53:08)_[M+NH_4_]^+^ | 878·7232 | 0·61 | 2·18 |  | 1·43 | 0·10 | 0·31 |  |
| TG(53:09)_[M+NH_4_]^+^ | 876·7076 | 0·31 | 0·02 | 0·92 | 3·30 |  | 0·00 | 0·11 |
| TG(54:00)_[M+NH_4_]^+^ | 908·8641 | 1·50 | 1·62 | 1·55 | 3·49 | 4·85 | 4·52 | 3·42 |
| TG(54:01)_[M+NH_4_]^+^ | 906·8484 | 0·02 |  |  |  | 0·94 | 0·89 | 1·09 |
| TG(54:03)_[M+NH_4_]^+^ | 902·8171 | 1·01 | 0·35 | 1·99 | 3·10 |  | 0·42 | 0·20 |
| TG(54:05)_[M+Na]^+^ | 903·7412 | 0·75 | 2·26 |  | 1·22 | 0·03 | 0·58 | 0·09 |
| TG(54:05)_[M+NH_4_]^+^ | 898·7858 | 1·75 | 6·99 |  | 3·12 |  | 2·05 |  |
| TG(54:08)_[M+NH_4_]^+^ | 892·7389 | 0·07 |  |  | 3·02 | 0·92 | 1·45 | 0·26 |
| TG(54:09)_[M+NH_4_]^+^ | 890·7232 | 0·94 | 0·11 |  | 2·52 | 2·71 |  | 0·89 |
| TG(54:10)_[M+NH_4_]^+^ | 888·7080 | 0·80 | 2·80 | 2·18 | 5·48 | 3·41 | 2·97 |  |
| TG(54:10)_[M+NH_4_]^+^ | 888·7076 | 0·80 | 2·80 | 2·18 | 5·48 | 3·41 | 2·97 |  |
| TG(54:11)_[M+NH_4_]^+^ | 886·6924 | 1·42 | 1·65 | 0·92 | 11·89 |  |  | 19·69 |
| TG(54:11)_[M+NH_4_]^+^ | 886·6919 | 1·42 | 1·65 | 0·92 | 11·89 |  |  | 19·69 |
| TG(54:12)_[M+NH_4_]^+^ | 884·6763 | 0·12 |  | 0·06 | 1·84 | 1·36 | 0·64 | 0·72 |
| TG(55:00)_[M+NH_4_]^+^ | 922·8797 | 3·55 | 1·75 | 2·75 | 3·51 | 3·13 | 3·96 | 4·93 |
| TG(55:01)_[M+NH_4_]^+^ | 920·8641 | 1·89 | 0·96 | 0·69 | 1·67 | 2·09 | 1·26 | 2·94 |
| TG(55:02)_[M+NH_4_]^+^ | 918·8484 | 0·70 | 0·72 | 0·44 | 0·26 | 0·87 | 0·74 |  |
| TG(55:03)_[M+NH_4_]^+^ | 916·8328 | 0·62 | 0·79 | 1·02 | 1·45 | 1·46 | 1·35 | 1·79 |
| TG(55:05)_[M+Na]^+^ | 917·7569 | 0·84 | 0·73 | 0·87 | 1·34 | 0·94 | 0·83 | 1·75 |
| TG(55:06)_[M+Na]^+^ | 915·7412 | 0·63 |  | 1·20 | 2·12 | 3·12 |  | 2·17 |
| TG(55:07)_[M+Na]^+^ | 913·7256 | 0·47 | 0·30 | 0·28 | 1·13 | 0·38 | 0·41 |  |
| TG(55:08)_[M+Na]^+^ | 911·7099 | 1·04 |  | 1·02 |  |  |  |  |
| TG(55:09)_[M+NH_4_]^+^ | 904·7389 | 0·56 | 2·01 | 0·38 | 1·07 | 1·10 | 1·19 | 1·00 |
| TG(55:10)_[M+NH_4_]^+^ | 902·7232 | 0·43 | 0·24 | 0·35 | 1·61 | 0·10 | 0·26 | 0·13 |
| TG(56:00)_[M+NH_4_]^+^ | 936·8954 | 5·65 | 8·98 | 10·30 | 11·62 | 12·00 | 12·42 | 14·35 |
| TG(56:03)_[M+NH_4_]^+^ | 930·8484 | 7·37 | 8·36 | 8·16 | 12·44 | 17·41 | 11·42 | 13·75 |
| TG(56:04)_[M+NH_4_]^+^ | 928·8328 | 6·06 | 7·49 | 9·74 | 11·42 | 10·66 | 9·50 | 11·03 |
| TG(56:06)_[M+Na]^+^ | 929·7569 | 6·61 | 9·30 | 10·54 | 14·60 | 16·33 | 14·75 |  |
| TG(56:07)_[M+Na]^+^ | 927·7412 | 5·82 | 8·65 |  | 12·10 | 9·75 | 8·69 |  |
| TG(56:08)_[M+Na]^+^ | 925·7256 | 4·83 | 5·04 | 6·15 | 6·04 |  |  |  |
| TG(56:10)_[M+Na]^+^ | 921·6943 | 2·69 | 0·78 |  | 3·16 |  | 1·21 | 3·17 |
| TG(56:10)_[M+NH_4_]^+^ | 916·7389 | 0·78 | 0·87 |  | 1·14 | 2·41 |  | 1·91 |
| TG(56:11)_[M+NH_4_]^+^ | 914·7232 | 0·51 | 0·48 | 0·56 | 0·93 | 1·37 |  | 1·73 |
| TG(56:12)_[M+NH_4_]^+^ | 912·7076 | 0·60 |  | 0·50 |  | 0·69 |  |  |
| TG(57:00)_[M+Na]^+^ | 955·8664 | 2·30 | 2·80 | 3·96 | 2·89 | 2·90 | 2·44 | 2·29 |
| TG(57:01)_[M+NH_4_]^+^ | 948·8954 | 0·73 | 1·19 | 0·97 | 1·54 | 2·00 | 1·07 | 1·64 |
| TG(57:02)_[M+NH_4_]^+^ | 946·8797 | 1·38 | 1·22 | 0·92 | 1·43 | 2·03 | 1·08 | 1·14 |
| TG(57:03)_[M+NH_4_]^+^ | 944·8641 | 1·46 | 0·92 | 1·31 |  | 3·60 | 2·25 | 2·37 |
| TG(57:05)_[M+Na]^+^ | 945·7882 | 2·49 | 1·89 | 1·43 | 1·53 | 2·14 | 1·53 | 1·90 |
| TG(57:06)_[M+Na]^+^ | 943·7725 | 1·43 | 3·09 | 2·75 | 4·45 | 6·31 | 5·22 | 6·68 |
| TG(57:07)_[M+Na]^+^ | 941·7569 |  | 12·07 | 12·53 |  |  | 15·58 |  |
| TG(57:08)_[M+Na]^+^ | 939·7412 | 7·38 | 10·31 |  | 10·45 | 13·85 |  | 8·01 |
| TG(57:08)_[M+NH_4_]^+^ | 934·7858 | 5·11 | 7·91 | 8·61 | 9·70 | 11·49 | 10·23 | 11·37 |
| TG(57:09)_[M+NH_4_]^+^ | 932·7702 | 11·66 | 15·06 | 21·08 | 25·27 | 24·83 | 23·13 | 21·73 |
| TG(57:10)_[M+Na]^+^ | 935·7099 | 4·63 | 5·97 | 6·46 | 6·61 |  | 8·01 |  |
| TG(57:10)_[M+NH_4_]^+^ | 930·7545 | 7·30 | 8·15 | 8·05 | 12·30 | 16·64 | 10·73 |  |
| TG(57:11)_[M+NH_4_]^+^ | 928·7389 | 6·27 | 7·28 |  | 12·63 |  |  |  |
| TG(57:12)_[M+NH_4_]^+^ | 926·7232 | 10·02 | 9·70 | 13·83 |  |  |  |  |
| TG(58:00)_[M+NH_4_]^+^ | 964·9267 | 4·56 | 7·69 | 13·32 | 5·32 | 2·04 | 7·38 | 0·55 |
| TG(58:01)_[M+Na]^+^ | 967·8664 | 1·22 | 0·62 | 0·49 | 0·38 | 0·34 | 0·48 | 0·36 |
| TG(58:01)_[M+NH_4_]^+^ | 962·9110 | 2·99 | 2·56 | 5·43 | 1·79 | 2·01 | 3·43 | 0·58 |
| TG(58:03)_[M+NH_4_]^+^ | 958·8797 | 2·44 | 1·98 | 3·75 | 2·13 | 1·84 | 1·67 | 1·11 |
| TG(58:05)_[M+Na]^+^ | 959·8038 | 3·45 | 3·44 | 6·97 | 3·26 | 2·97 | 2·74 | 1·76 |
| TG(58:06)_[M+NH_4_]^+^ | 952·8328 | 2·48 | 3·52 | 3·72 | 4·12 | 4·27 | 3·30 | 2·93 |
| TG(58:07)_[M+Na]^+^ | 955·7725 | 2·26 | 2·97 | 4·19 | 3·29 | 3·15 | 2·22 |  |
| TG(58:08)_[M+Na]^+^ | 953·7569 | 2·86 | 4·18 |  | 4·89 | 4·96 |  |  |
| TG(58:09)_[M+Na]^+^ | 951·7412 | 2·00 | 2·82 | 3·06 | 3·42 | 3·96 |  |  |
| TG(58:09)_[M+NH_4_]^+^ | 946·7858 | 1·52 | 0·99 | 0·99 | 1·51 | 2·06 | 1·14 | 1·72 |
| TG(58:10)_[M+Na]^+^ | 949·7256 | 1·14 | 1·63 | 1·39 | 1·92 | 2·22 |  | 1·72 |
| TG(58:10)_[M+NH_4_]^+^ | 944·7702 | 1·19 | 2·05 | 1·35 | 2·48 | 3·89 | 2·46 | 2·76 |
| TG(58:11)_[M+NH_4_]^+^ | 942·7545 | 4·23 |  | 7·09 | 7·84 | 10·18 |  |  |
| TG(58:14)_[M+Na]^+^ | 941·6630 | 8·50 | 15·38 | 15·28 | 15·76 | 19·48 | 19·71 | 17·06 |
| TG(58:14)_[M+NH_4_]^+^ | 936·7076 | 4·75 | 8·49 |  | 9·28 | 11·83 | 11·12 | 14·04 |
| TG(59:00)_[M+Na]^+^ | 983·8977 | 0·16 | 0·18 | 0·25 | 0·25 | 0·26 |  | 0·29 |
| TG(59:00)_[M+NH_4_]^+^ | 978·9423 | 0·77 | 0·55 | 0·57 | 0·58 | 0·42 | 0·75 | 0·38 |
| TG(59:01)_[M+Na]^+^ | 981·8821 | 0·07 | 0·03 | 0·11 | 0·03 | 0·03 | 0·01 | 0·13 |
| TG(59:02)_[M+NH_4_]^+^ | 974·9110 | 0·20 | 0·30 | 0·25 | 0·40 | 0·31 | 0·35 | 0·24 |
| TG(59:03)_[M+NH_4_]^+^ | 972·8954 | 0·33 | 0·55 | 0·30 | 0·27 | 0·22 | 0·40 | 0·34 |
| TG(59:04)_[M+NH_4_]^+^ | 970·8797 | 0·42 | 0·97 | 0·32 | 0·29 | 0·26 | 0·32 | 0·36 |
| TG(59:05)_[M+NH_4_]^+^ | 968·8641 | 0·76 | 0·64 | 0·32 | 0·40 | 0·43 | 0·42 | 0·43 |
| TG(59:06)_[M+Na]^+^ | 971·8038 | 0·37 | 0·70 | 0·28 | 0·27 | 0·19 | 0·28 | 0·30 |
| TG(59:06)_[M+NH_4_]^+^ | 966·8489 | 1·05 | 1·07 | 1·98 | 0·98 | 0·43 | 1·51 | 0·31 |
| TG(59:07)_[M+Na]^+^ | 969·7882 | 0·46 | 1·26 | 0·35 | 0·41 | 0·29 | 0·51 | 0·35 |
| TG(59:08)_[M+Na]^+^ | 967·7725 | 1·92 | 0·63 |  |  |  |  | 0·34 |
| TG(59:09)_[M+Na]^+^ | 965·7569 | 3·10 | 4·96 | 8·89 |  |  | 2·73 |  |
| TG(59:09)_[M+NH_4_]^+^ | 960·8015 | 2·49 | 2·46 | 4·96 | 2·31 | 2·04 | 1·99 | 1·22 |
| TG(59:10)_[M+Na]^+^ | 963·7412 | 13·62 | 7·96 | 36·25 | 2·18 |  | 40·88 | 0·96 |
| TG(59:10)_[M+NH_4_]^+^ | 958·7863 | 2·30 | 2·03 | 3·28 | 1·52 | 1·68 | 1·06 | 1·02 |
| TG(59:11)_[M+Na]^+^ | 961·7256 | 2·04 | 2·16 | 5·62 |  |  | 2·49 | 0·72 |
| TG(59:11)_[M+NH_4_]^+^ | 956·7702 | 1·38 | 1·63 | 2·73 | 1·45 |  |  |  |
| TG(60:00)_[M+NH_4_]^+^ | 992·9580 | 0·36 | 0·24 | 0·20 | 0·21 | 0·21 | 0·18 | 0·21 |
| TG(60:01)_[M+NH_4_]^+^ | 990·9423 | 0·20 | 0·17 | 0·21 | 0·13 | 0·18 | 0·14 | 0·23 |
| TG(60:02)_[M+Na]^+^ | 993·8821 | 0·30 | 0·32 | 0·09 | 0·08 | 0·17 | 0·06 | 0·13 |
| TG(60:02)_[M+NH_4_]^+^ | 988·9267 | 0·22 | 0·23 | 0·21 | 0·22 | 0·38 | 0·24 | 0·25 |
| TG(60:03)_[M+NH_4_]^+^ | 986·9110 | 0·20 | 0·31 | 0·45 | 0·37 | 0·46 | 0·33 | 0·26 |
| TG(60:04)_[M+NH_4_]^+^ | 984·8954 | 0·19 | 0·29 | 0·49 | 0·34 | 0·26 | 0·49 | 0·26 |
| TG(60:05)_[M+Na]^+^ | 987·8351 | 0·15 | 0·25 | 0·31 | 0·30 | 0·40 | 0·28 | 0·26 |
| TG(60:05)_[M+NH_4_]^+^ | 982·8797 | 0·11 | 0·10 | 0·14 | 0·13 | 0·14 | 0·11 | 0·20 |
| TG(60:06)_[M+Na]^+^ | 985·8195 | 0·29 | 0·49 | 0·81 | 0·58 | 0·48 | 0·54 | 0·31 |
| TG(60:07)_[M+Na]^+^ | 983·8038 | 0·15 | 0·15 | 0·19 | 0·17 |  | 0·13 | 0·26 |
| TG(60:08)_[M+Na]^+^ | 981·7882 | 0·07 | 0·02 | 0·02 |  |  |  | 0·08 |
| TG(60:09)_[M+Na]^+^ | 979·7725 |  | 0·66 |  |  | 0·26 |  | 0·47 |
| TG(60:10)_[M+Na]^+^ | 977·7569 | 0·87 | 1·92 | 1·07 | 1·88 | 0·69 |  | 0·31 |
| TG(60:10)_[M+NH_4_]^+^ | 972·8015 | 0·40 | 0·59 | 0·23 | 0·34 | 0·31 | 0·33 | 0·34 |
| TG(60:11)_[M+Na]^+^ | 975·7412 | 0·53 |  | 0·30 | 0·40 | 0·53 | 0·63 |  |
| TG(60:11)_[M+NH_4_]^+^ | 970·7863 | 0·30 | 1·57 | 0·31 | 0·37 |  | 0·32 | 0·37 |
| TG(60:11)_[M+NH_4_]^+^ | 970·7858 | 0·30 | 1·57 | 0·31 | 0·37 |  | 0·32 | 0·37 |
| TG(60:12)_[M+Na]^+^ | 973·7256 | 0·34 | 0·68 | 0·37 | 0·29 |  |  |  |
| TG(60:12)_[M+NH_4_]^+^ | 968·7706 | 1·20 | 0·34 | 0·28 | 0·35 | 0·27 | 0·44 |  |
| TG(60:12)_[M+NH_4_]^+^ | 968·7702 | 1·20 | 0·34 | 0·28 | 0·35 | 0·27 | 0·44 |  |
| TG(60:13)_[M+NH_4_]^+^ | 966·7545 | 0·82 | 1·19 | 2·22 |  |  |  |  |
| TG(60:14)_[M+NH_4_]^+^ | 964·7389 |  | 15·92 | 26·71 | 10·61 |  |  | 0·57 |
| TG(60:15)_[M+Na]^+^ | 967·6786 | 1·27 | 0·55 | 0·47 | 0·29 | 0·23 | 0·41 | 0·25 |
| TG(60:15)_[M+NH_4_]^+^ | 962·7232 | 2·31 | 2·22 | 6·67 | 0·81 |  | 2·49 | 0·54 |
| TG(61:00)_[M+Na]^+^ | 1011·9290 | 0·22 | 0·12 | 0·22 |  |  | 0·22 |  |
| TG(61:00)_[M+NH_4_]^+^ | 1006·9741 | 0·20 | 0·31 | 0·39 | 0·15 | 0·28 | 0·55 | 0·19 |
| TG(61:00)_[M+NH_4_]^+^ | 1006·9736 | 0·20 | 0·31 | 0·39 | 0·15 | 0·28 | 0·55 | 0·19 |
| TG(61:01)_[M+NH_4_]^+^ | 1004·9580 | 0·25 | 0·22 | 0·33 | 0·24 | 0·28 | 0·15 | 0·22 |
| TG(61:03)_[M+Na]^+^ | 1005·8821 | 0·32 | 0·35 | 0·46 | 0·24 | 0·29 | 0·18 | 0·22 |
| TG(61:05)_[M+NH_4_]^+^ | 996·8954 |  | 0·02 |  |  | 0·11 | 0·02 | 0·02 |
| TG(61:06)_[M+NH_4_]^+^ | 994·8797 | 0·18 | 0·25 | 0·02 | 0·04 | 0·09 | 0·03 | 0·11 |
| TG(61:07)_[M+NH_4_]^+^ | 992·8641 | 0·36 | 0·33 | 0·21 | 0·22 | 0·22 | 0·19 | 0·22 |
| TG(61:10)_[M+Na]^+^ | 991·7725 | 0·24 | 0·22 | 0·24 |  | 0·20 |  | 0·17 |
| TG(61:10)_[M+NH_4_]^+^ | 986·8171 | 0·19 | 0·32 | 0·48 | 0·37 | 0·37 | 0·34 | 0·25 |
| TG(61:11)_[M+Na]^+^ | 989·7569 | 0·25 | 0·18 | 0·21 | 0·19 | 0·28 | 0·18 | 0·24 |
| TG(61:11)_[M+NH_4_]^+^ | 984·8015 | 0·17 | 0·13 | 0·43 | 0·30 |  |  | 0·23 |
| TG(61:12)_[M+Na]^+^ | 987·7412 | 0·17 | 0·27 | 0·39 | 0·34 | 0·42 | 0·34 | 0·27 |
| TG(62:00)_[M+Na]^+^ | 1025·9447 | 0·52 | 0·28 | 0·50 |  |  | 0·32 |  |
| TG(62:00)_[M+NH_4_]^+^ | 1020·9893 | 0·33 | 0·17 | 0·39 | 0·23 | 0·72 | 0·18 |  |
| TG(62:01)_[M+Na]^+^ | 1023·9290 | 0·35 | 0·19 | 0·35 | 0·19 |  | 0·25 |  |
| TG(62:01)_[M+NH_4_]^+^ | 1018·9736 | 0·34 | 0·14 | 0·32 | 0·17 | 0·49 | 0·14 | 0·28 |
| TG(62:02)_[M+Na]^+^ | 1021·9134 | 0·40 | 0·17 | 0·39 | 0·20 | 0·56 | 0·19 | 0·32 |
| TG(62:02)_[M+NH_4_]^+^ | 1016·9580 | 0·54 | 0·30 | 0·48 | 0·36 | 0·60 |  |  |
| TG(62:03)_[M+Na]^+^ | 1019·8977 | 0·36 | 0·13 | 0·32 | 0·14 | 0·48 | 0·14 | 0·28 |
| TG(62:03)_[M+NH_4_]^+^ | 1014·9423 | 0·36 | 0·14 | 0·35 | 0·25 |  | 0·20 |  |
| TG(62:04)_[M+NH_4_]^+^ | 1012·9267 | 0·24 | 0·14 | 0·26 | 0·30 | 0·42 | 0·30 | 0·37 |
| TG(62:05)_[M+NH_4_]^+^ | 1010·9110 | 0·11 |  | 0·13 | 0·20 | 0·39 | 0·18 | 0·21 |
| TG(62:06)_[M+Na]^+^ | 1013·8508 | 0·25 | 0·14 | 0·23 | 0·24 | 0·41 | 0·21 | 0·29 |
| TG(62:06)_[M+NH_4_]^+^ | 1008·8954 |  | 0·11 | 0·06 | 0·03 | 0·27 | 0·16 | 0·12 |
| TG(62:07)_[M+Na]^+^ | 1011·8351 | 0·25 | 0·17 | 0·26 | 0·33 | 0·43 | 0·32 | 0·31 |
| TG(62:07)_[M+NH_4_]^+^ | 1006·8802 | 0·19 | 0·22 | 0·41 | 0·59 | 0·45 | 0·39 | 0·19 |
| TG(62:08)_[M+Na]^+^ | 1009·8195 | 0·26 |  | 0·33 | 0·40 | 0·53 | 0·43 | 0·29 |
| TG(62:08)_[M+NH_4_]^+^ | 1004·8641 | 0·27 | 0·31 | 0·33 | 0·24 | 0·24 | 0·21 | 0·24 |
| TG(62:09)_[M+Na]^+^ | 1007·8038 | 0·18 | 0·31 |  |  | 0·17 | 0·27 |  |
| TG(62:11)_[M+Na]^+^ | 1003·7725 | 0·21 | 0·30 | 0·12 | 0·07 | 0·20 |  | 0·39 |
| TG(62:15)_[M+Na]^+^ | 995·7099 | 0·09 | 0·09 | 0·01 | 0·05 | 0·12 | 0·04 | 0·12 |
| TG(62:15)_[M+NH_4_]^+^ | 990·7545 | 0·15 | 0·09 | 0·15 | 0·11 |  | 0·10 | 0·21 |
| TG(62:16)_[M+NH_4_]^+^ | 988·7389 | 0·24 | 0·25 | 0·24 | 0·23 | 0·31 |  | 0·26 |
| TG(63:00)_[M+Na]^+^ | 1039·9603 | 1·22 | 1·00 | 1·24 |  |  | 0·84 |  |
| TG(63:00)_[M+NH_4_]^+^ | 1035·0049 | 1·04 | 0·52 | 1·20 | 1·02 | 1·40 |  |  |
| TG(63:01)_[M+Na]^+^ | 1037·9447 | 1·17 | 0·80 | 0·99 |  |  |  |  |
| TG(63:01)_[M+NH_4_]^+^ | 1032·9893 | 0·85 | 0·32 | 1·05 | 0·93 | 1·12 |  |  |
| TG(63:02)_[M+Na]^+^ | 1035·9290 | 1·18 | 0·81 | 1·12 | 1·14 | 1·59 | 0·51 |  |
| TG(63:02)_[M+NH_4_]^+^ | 1030·9736 | 0·65 | 0·27 | 0·95 | 0·62 | 0·81 |  | 0·34 |
| TG(63:03)_[M+Na]^+^ | 1033·9134 | 0·96 | 0·63 | 0·94 | 0·94 | 1·35 | 0·39 | 0·50 |
| TG(63:03)_[M+NH_4_]^+^ | 1028·9580 | 0·74 | 0·19 | 0·61 |  | 0·79 |  |  |
| TG(63:04)_[M+NH_4_]^+^ | 1026·9423 | 0·69 | 0·31 | 0·74 | 0·34 |  | 0·26 |  |
| TG(63:05)_[M+Na]^+^ | 1029·8821 | 0·72 | 0·40 | 0·71 | 0·60 | 0·94 | 0·30 | 0·40 |
| TG(63:05)_[M+NH_4_]^+^ | 1024·9267 | 0·44 | 0·30 | 0·36 | 0·30 |  |  |  |
| TG(63:06)_[M+Na]^+^ | 1027·8664 | 0·63 | 0·36 | 0·65 | 0·53 | 0·77 | 0·30 | 0·45 |
| TG(63:06)_[M+NH_4_]^+^ | 1022·9110 | 0·38 |  | 0·30 | 0·03 | 0·21 |  | 0·17 |
| TG(63:07)_[M+Na]^+^ | 1025·8508 | 0·50 | 0·40 | 0·48 | 0·26 | 0·78 | 0·25 | 0·28 |
| TG(63:07)_[M+NH_4_]^+^ | 1020·8954 | 0·40 | 0·16 | 0·38 | 0·18 | 0·72 | 0·18 | 0·32 |
| TG(63:09)_[M+NH_4_]^+^ | 1016·8641 | 0·51 | 0·32 | 0·42 | 0·38 | 0·62 | 0·22 | 0·33 |
| TG(63:10)_[M+NH_4_]^+^ | 1014·8484 | 0·28 | 0·17 | 0·26 | 0·26 | 0·59 | 0·20 | 0·28 |
| TG(63:11)_[M+NH_4_]^+^ | 1012·8328 | 0·22 | 0·17 | 0·21 | 0·33 | 0·44 | 0·28 |  |
| TG(63:12)_[M+Na]^+^ | 1015·7725 | 0·42 | 0·21 |  | 0·40 |  |  | 0·41 |
| TG(63:12)_[M+NH_4_]^+^ | 1010·8171 | 0·16 |  | 0·20 | 0·27 |  |  | 0·19 |
| TG(63:13)_[M+Na]^+^ | 1013·7569 |  | 0·16 | 0·19 | 0·28 | 0·31 | 0·23 | 0·31 |
| TG(64:01)_[M+Na]^+^ | 1051·9603 | 0·14 | 0·08 | 0·08 |  |  | 0·20 |  |
| TG(64:01)_[M+NH_4_]^+^ | 1047·0049 | 0·16 | 0·10 | 0·14 | 0·16 | 0·20 | 0·15 |  |
| TG(64:02)_[M+NH_4_]^+^ | 1044·9893 | 0·51 | 0·53 | 0·47 | 0·71 | 0·68 | 0·34 |  |
| TG(64:03)_[M+Na]^+^ | 1047·9290 | 0·10 | 0·10 | 0·08 | 0·13 | 0·14 | 0·18 |  |
| TG(64:03)_[M+NH_4_]^+^ | 1042·9736 | 1·61 | 1·05 | 1·31 | 2·62 | 1·95 |  |  |
| TG(64:04)_[M+Na]^+^ | 1045·9134 | 0·36 | 0·28 | 0·26 | 0·38 | 0·36 | 0·27 |  |
| TG(64:04)_[M+NH_4_]^+^ | 1040·9580 | 1·29 | 1·13 | 1·18 |  | 1·59 | 0·80 |  |
| TG(64:05)_[M+NH_4_]^+^ | 1038·9423 | 1·30 | 0·89 | 1·06 |  | 1·70 | 0·72 |  |
| TG(64:06)_[M+Na]^+^ | 1041·8821 | 1·19 | 1·34 | 1·26 | 2·05 | 1·76 | 0·78 |  |
| TG(64:06)_[M+NH_4_]^+^ | 1036·9267 | 1·37 | 0·95 | 0·99 | 1·27 | 1·66 | 0·41 |  |
| TG(64:07)_[M+Na]^+^ | 1039·8664 | 1·35 | 1·07 | 1·21 | 1·69 | 1·77 | 0·81 |  |
| TG(64:07)_[M+NH_4_]^+^ | 1034·9110 | 1·02 | 0·66 | 0·97 | 1·00 | 1·43 | 0·42 |  |
| TG(64:08)_[M+Na]^+^ | 1037·8508 | 1·40 | 0·99 | 1·24 | 1·33 |  | 0·41 |  |
| TG(64:09)_[M+NH_4_]^+^ | 1030·8797 | 0·77 | 0·43 | 0·75 | 0·61 | 0·99 | 0·29 | 0·46 |
| TG(64:10)_[M+Na]^+^ | 1033·8195 | 1·02 | 0·46 | 0·70 | 1·02 |  | 0·46 |  |
| TG(64:10)_[M+NH_4_]^+^ | 1028·8641 | 0·61 | 0·32 | 0·59 | 0·45 | 0·77 | 0·23 | 0·33 |
| TG(64:11)_[M+NH_4_]^+^ | 1026·8484 | 0·57 | 0·30 | 0·43 | 0·32 | 0·77 | 0·26 | 0·27 |
| TG(64:12)_[M+Na]^+^ | 1029·7882 | 0·86 | 0·40 | 0·57 | 0·55 | 1·19 | 0·31 | 0·40 |
| TG(64:12)_[M+NH_4_]^+^ | 1024·8328 | 0·54 | 0·40 |  | 0·33 | 0·97 |  |  |
| TG(64:13)_[M+Na]^+^ | 1027·7725 | 0·63 | 0·35 | 0·49 |  | 1·07 | 0·30 | 0·36 |
| TG(64:13)_[M+NH_4_]^+^ | 1022·8171 | 0·35 |  |  |  | 0·25 |  | 0·20 |
| TG(64:14)_[M+Na]^+^ | 1025·7569 | 0·47 | 0·25 | 0·45 | 0·22 | 1·12 | 0·23 | 0·34 |
| TG(64:15)_[M+NH_4_]^+^ | 1018·7858 | 0·34 |  | 0·22 | 0·18 |  |  | 0·19 |
| TG(64:16)_[M+Na]^+^ | 1021·7256 | 0·41 | 0·17 | 0·40 | 0·24 | 0·58 | 0·21 | 0·36 |
| TG(65:00)_[M+Na]^+^ | 1067·9916 | 0·35 | 0·72 | 0·75 | 0·29 | 0·48 |  |  |
| TG(65:00)_[M+NH_4_]^+^ | 1063·0362 | 1·07 | 1·69 | 1·46 | 0·28 | 0·62 |  |  |
| TG(65:01)_[M+NH_4_]^+^ | 1061·0206 | 0·55 | 0·45 | 0·51 |  | 0·59 | 0·22 |  |
| TG(65:02)_[M+Na]^+^ | 1063·9603 | 0·32 | 0·90 | 0·70 | 0·21 |  |  |  |
| TG(65:02)_[M+NH_4_]^+^ | 1059·0049 | 0·43 | 0·17 | 0·28 | 0·35 | 0·47 |  |  |
| TG(65:03)_[M+NH_4_]^+^ | 1056·9893 | 0·18 |  | 0·16 | 0·03 |  |  |  |
| TG(65:04)_[M+Na]^+^ | 1059·9290 | 0·59 | 0·32 | 0·43 | 0·54 | 0·59 | 0·30 |  |
| TG(65:04)_[M+NH_4_]^+^ | 1054·9736 | 0·20 | 0·02 | 0·02 | 0·04 |  |  |  |
| TG(65:05)_[M+Na]^+^ | 1057·9134 | 0·21 |  | 0·06 | 0·12 | 0·25 | 0·03 |  |
| TG(65:05)_[M+NH_4_]^+^ | 1052·9580 |  | 0·05 | 0·14 |  | 0·15 |  |  |
| TG(65:06)_[M+Na]^+^ | 1055·8977 | 0·33 | 0·07 | 0·24 | 0·18 | 0·23 | 0·10 |  |
| TG(65:08)_[M+Na]^+^ | 1051·8664 | 0·10 |  | 0·31 | 0·05 | 0·14 | 0·14 |  |
| TG(65:08)_[M+NH_4_]^+^ | 1046·9110 | 0·17 | 0·15 | 0·15 | 0·23 | 0·21 | 0·24 |  |
| TG(65:09)_[M+NH_4_]^+^ | 1044·8954 | 0·58 | 0·60 | 0·52 | 0·78 | 0·74 | 0·36 |  |
| TG(65:10)_[M+NH_4_]^+^ | 1042·8797 | 1·79 | 1·72 | 1·54 | 2·23 | 1·95 | 0·66 |  |
| TG(65:11)_[M+NH_4_]^+^ | 1040·8641 | 1·28 | 1·31 | 1·22 | 1·61 | 1·63 | 0·79 |  |
| TG(65:12)_[M+Na]^+^ | 1043·8038 | 1·31 | 1·14 | 1·05 | 1·41 |  | 0·52 |  |
| TG(65:12)_[M+NH_4_]^+^ | 1038·8484 | 1·36 |  | 1·13 | 1·83 |  | 0·49 |  |
| TG(66:01)_[M+Na]^+^ | 1079·9916 |  | 0·62 | 0·30 |  | 0·18 |  |  |
| TG(66:04)_[M+NH_4_]^+^ | 1068·9893 | 0·31 | 0·55 | 0·56 | 0·42 | 0·34 |  |  |
| TG(66:06)_[M+Na]^+^ | 1069·9134 | 0·04 | 0·12 | 0·10 | 0·98 | 0·31 |  |  |
| TG(66:06)_[M+NH_4_]^+^ | 1064·9580 | 0·36 | 0·51 | 0·59 |  | 0·53 | 0·08 |  |
| TG(66:07)_[M+Na]^+^ | 1067·8977 |  | 0·43 | 0·40 | 0·24 | 0·77 |  |  |
| TG(66:07)_[M+NH_4_]^+^ | 1062·9423 | 0·37 | 1·32 | 0·84 | 0·16 | 0·38 |  |  |
| TG(66:08)_[M+Na]^+^ | 1065·8821 | 0·44 | 0·36 | 0·35 | 0·48 | 0·60 |  |  |
| TG(66:08)_[M+NH_4_]^+^ | 1060·9267 | 0·55 | 0·45 | 0·52 | 0·54 | 0·55 | 0·23 |  |
| TG(66:11)_[M+Na]^+^ | 1059·8351 | 0·59 | 0·31 | 0·40 |  |  |  |  |
| TG(66:11)_[M+NH_4_]^+^ | 1054·8797 | 0·14 | 0·07 | 0·06 | 0·10 | 0·10 |  |  |
| TG(66:12)_[M+Na]^+^ | 1057·8195 | 0·19 |  | 0·12 | 0·29 | 0·28 |  |  |
| TG(66:12)_[M+NH_4_]^+^ | 1052·8641 |  | 0·17 | 0·20 | 0·08 | 0·17 | 0·14 |  |
| TG(66:15)_[M+Na]^+^ | 1051·7725 |  | 0·13 |  | 0·05 |  | 0·22 |  |
| TG(66:16)_[M+Na]^+^ | 1049·7569 | 0·04 | 0·04 |  |  | 0·06 | 0·22 |  |
| TG(66:17)_[M+Na]^+^ | 1047·7412 | 0·09 | 0·09 | 0·07 | 0·13 | 0·13 | 0·18 |  |
| TG(66:17)_[M+NH_4_]^+^ | 1042·7858 | 2·14 | 1·99 | 1·79 | 2·46 | 2·14 | 0·76 |  |
| TG(66:18)_[M+Na]^+^ | 1045·7256 | 0·43 | 0·36 | 0·30 | 0·46 | 0·40 | 0·29 |  |
| TG(66:18)_[M+NH_4_]^+^ | 1040·7702 | 1·36 | 0·83 | 1·06 | 1·72 |  | 0·78 |  |
| TG(67:01)_[M+NH_4_]^+^ | 1089·0523 | 0·40 | 0·20 | 0·18 | 0·40 | 0·34 |  |  |
| TG(67:03)_[M+NH_4_]^+^ | 1085·0210 | 0·35 | 0·15 | 0·13 | 0·34 | 0·27 |  |  |
| TG(67:11)_[M+NH_4_]^+^ | 1068·8958 | 0·24 | 0·40 | 0·74 | 0·40 | 0·63 |  |  |
| TG(68:12)_[M+NH_4_]^+^ | 1080·8958 | 0·26 | 0·58 | 0·18 | 0·20 | 0·31 |  |  |

*Table S5. Signal intensities for high resolution* m/z *values consistent with triglyceride isoforms (n = 2). Signals were assigned according to high resolution masses with a deviation of <12 ppm from the calculated monoisotopic mass. Quantitative standards were not used. G, guanidinium. Collection points: +2 h, early part of G_1_; +4 h, mid-G_1_ (first CP); +6 h, end of G_1_ and pS; +8 h, S and second CP; +10 h, G_2_; +13 h, M; +15 h, G_3_, for others see* Figure 1*.*

|  |  | Collection point | | | | | | |
| --- | --- | --- | --- | --- | --- | --- | --- | --- |
| Sterol annotation | *m/z* | +2 h | +4h | +6 h | +8 h | +10 h | +13 h | +15 h |
| 16:0 Campesteryl ester_[M+H]^+^ | 639·608 | 0·02 | 0·14 | 0·06 | 0·71 | 0·42 | 0·45 | 1·31 |
| 16:0 Campesteryl ester_[M+K]^+^ | 677·5634 | 1·59 | 2·20 | 2·31 | 1·40 | 1·17 | 1·22 | 1·25 |
| 16:0 Campesteryl ester_[M+NH_4_]^+^ | 656·634 | 0·32 |  | 0·69 | 0·80 | 1·01 |  | 1·38 |
| 16:0 Sitosteryl ester_[M+H]^+^ | 653·6236 | 0·18 | 0·13 |  | 0·07 |  |  |  |
| 16:0 Sitosteryl ester_[M+Na]^+^ | 675·6056 | 1·08 | 0·85 | 0·32 | 0·24 | 0·36 | 0·42 | 1·23 |
| 16:0 Stigmasteryl ester/16:1 Sitosteryl ester/16:0 Brassicasteryl ester_[M+G]^+^ | 745·6252 |  | 0·31 |  | 0·98 | 1·67 | 2·02 | 3·70 |
| 16:0 Stigmasteryl ester/16:1 Sitosteryl ester/16:0 Brassicasteryl ester_[M+Na]^+^ | 673·59 | 0·22 | 0·49 | 0·87 | 0·65 | 0·22 | 0·35 | 0·67 |
| 16:0 Stigmasteryl ester/16:1 Sitosteryl ester/16:0 Brassicasteryl ester_[M+NH_4_]^+^ | 668·634 | 0·25 | 0·30 |  | 0·11 | 0·11 | 0·17 | 0·28 |
| 16:1 Campesteryl ester_[M+G]^+^ | 731·6095 | 4·89 | 2·37 |  | 2·57 | 9·34 | 10·19 | 27·21 |
| 16:1 Campesteryl ester_[M+H]^+^ | 637·5923 | 0·11 | 0·27 |  | 0·39 | 0·12 | 0·21 | 0·38 |
| 16:1 Campesteryl ester_[M+K]^+^ | 675·5477 | 0·84 | 0·61 | 0·08 | 0·05 | 0·12 | 0·21 | 1·06 |
| 16:1 Stigmasteryl ester/16:2 Sitosteryl ester_[M+G]^+^ | 743·6095 | 1·35 | 1·98 | 1·70 | 4·55 | 4·58 | 3·28 | 4·33 |
| 16:1 Stigmasteryl ester/16:2 Sitosteryl ester_[M+Na]^+^ | 671·5743 | 0·07 | 0·25 | 0·16 | 0·13 | 0·20 | 0·12 |  |
| 16:2 Campesteryl ester_[M+G]^+^ | 729·5939 | 16·78 | 11·96 | 9·42 | 10·98 | 16·91 | 18·71 | 25·39 |
| 16:2 Campesteryl ester_[M+H]^+^ | 635·5767 | 0·13 | 0·53 | 0·34 | 0·52 | 0·19 | 0·08 | 0·72 |
| 16:2 Stigmasteryl ester/16:3 Sitosteryl ester_[M+G]^+^ | 741·5939 | 3·33 | 3·96 | 5·17 | 8·88 | 6·99 | 4·40 | 3·70 |
| 16:2 Stigmasteryl ester/16:3 Sitosteryl ester_[M+H]^+^ | 647·5767 | 0·58 | 0·17 | 0·16 | 0·24 | 0·18 | 0·15 | 0·18 |
| 16:3 Campesteryl ester/16:0 ergosteryl ester_[M+G]^+^ | 727·5782 | 41·56 | 34·30 | 23·66 | 16·99 | 28·99 | 34·08 | 35·77 |
| 16:3 Stigmasteryl ester_[M+G]^+^ | 739·5782 | 4·39 | 5·52 | 5·41 | 12·92 | 13·00 | 9·93 | 11·89 |
| 16:3 Stigmasteryl ester_[M+K]^+^ | 683·5164 | 0·93 | 1·18 | 2·05 | 0·51 | 0·37 | 1·03 | 0·26 |
| 18:0 Campesteryl ester_[M+H]^+^ | 667·6393 | 0·42 | 0·36 | 0·45 | 0·37 | 0·22 | 0·35 | 0·30 |
| 18:0 Campesteryl ester_[M+K]^+^ | 705·5947 | 37·00 | 43·13 |  |  |  | 32·73 |  |
| 18:0 Campesteryl ester_[M+Na]^+^ | 689·6213 | 0·16 | 0·32 |  | 0·10 |  | 0·03 | 0·23 |
| 18:0 Campesteryl ester_[M+NH_4_]^+^ | 684·6653 | 2·00 |  | 3·97 | 1·84 |  | 3·33 | 0·59 |
| 18:0 Sitosteryl ester_[M+H]^+^ | 681·6549 | 0·42 | 0·05 | 0·66 | 0·48 |  | 0·30 | 0·21 |
| 18:0 Sitosteryl ester_[M+K]^+^ | 719·6103 | 3·83 | 2·77 | 3·11 |  |  |  | 4·98 |
| 18:0 Sitosteryl ester_[M+Na]^+^ | 703·6369 | 5·16 | 6·60 | 5·51 | 7·23 | 7·12 | 8·32 | 5·99 |
| 18:1 Campesteryl ester/18:0 Brassicasteryl ester_[M+G]^+^ | 759·6408 | 17·27 | 20·01 | 15·91 | 44·84 | 49·51 | 44·52 | 51·49 |
| 18:1 Campesteryl ester/18:0 Brassicasteryl ester_[M+K]^+^ | 703·579 | 4·13 | 5·88 | 3·34 | 7·46 |  |  |  |
| 18:1 Campesteryl ester/18:0 Brassicasteryl ester_[M+Na]^+^ | 687·6056 | 0·16 | 0·45 | 0·57 | 0·27 | 0·22 | 0·31 | 0·31 |
| 18:1 Campesteryl ester/18:0 Brassicasteryl ester_[M+NH_4_]^+^ | 682·6496 | 0·61 |  | 1·12 | 0·39 |  |  |  |
| 18:1 Sitosteryl ester/18:0 Stigmasteryl ester_[M+K]^+^ | 717·5947 | 2·25 | 3·91 | 1·59 | 3·29 | 2·93 | 1·13 | 1·46 |
| 18:1 Stigmasteryl ester/18:2 Sitosteryl ester_[M+G]^+^ | 771·6408 | 12·72 | 13·42 | 14·51 | 22·12 | 16·62 | 14·44 | 6·92 |
| 18:1 Stigmasteryl ester/18:2 Sitosteryl ester_[M+H]^+^ | 677·6236 | 1·63 | 1·99 | 1·69 |  | 1·21 | 1·51 | 1·15 |
| 18:1 Stigmasteryl ester/18:2 Sitosteryl ester_[M+Na]^+^ | 699·6056 | 0·65 | 1·37 | 1·38 | 1·21 | 0·85 | 0·82 |  |
| 18:2 Stigmasteryl ester/18:3 Sitosteryl ester_[M+G]^+^ | 769·6252 | 30·59 | 28·31 | 33·72 | 35·78 | 25·78 | 23·07 | 8·36 |
| 18:2 Stigmasteryl ester/18:3 Sitosteryl ester_[M+H]^+^ | 675·608 | 1·26 | 1·04 | 0·57 | 0·50 | 0·57 | 0·63 | 1·36 |
| 18:3 Campesteryl ester/18:1 ergosteryl ester/18:2 Brassicasteryl ester_[M+G]^+^ | 755·6095 | 68·06 | 48·43 | 42·25 | 38·77 | 39·35 | 40·54 | 53·81 |
| 18:3 Campesteryl ester/18:1 ergosteryl ester/18:2 Brassicasteryl ester_[M+Na]^+^ | 683·5743 | 1·24 | 1·46 | 2·42 | 0·86 | 0·57 | 1·29 | 0·40 |
| 18:3 Campesteryl ester/18:1 ergosteryl ester/18:2 Brassicasteryl ester_[M+NH_4_]^+^ | 678·6183 | 1·45 | 1·22 | 1·45 | 1·82 |  | 1·59 |  |
| 18:3 Stigmasteryl ester_[M+G]^+^ | 767·6095 | 47·99 | 49·95 | 69·38 | 69·05 | 72·65 | 47·57 | 20·67 |
| 18:3 Stigmasteryl ester_[M+H]^+^ | 673·5923 | 0·13 | 0·38 | 0·73 | 0·50 | 0·11 | 0·25 | 0·60 |
| 18:3 Stigmasteryl ester_[M+K]^+^ | 711·5477 | 2·36 | 2·39 | 2·94 | 3·97 | 4·13 | 4·18 | 6·34 |
| 20:0 Campesteryl ester_[M+Na]^+^ | 717·6526 | 2·11 | 3·72 | 1·26 | 1·29 | 2·71 | 2·72 | 1·33 |
| 20:0 Sitosteryl ester_[M+H]^+^ | 709·6862 | 2·27 | 1·50 | 1·59 |  | 2·74 |  | 4·48 |
| 20:0 Sitosteryl ester_[M+Na]^+^ | 731·6682 | 8·63 | 7·18 | 4·41 | 6·50 | 15·15 | 14·43 | 26·96 |
| 20:0 Stigmasteryl ester/20:1 Sitosteryl ester_[M+H]^+^ | 707·6706 |  | 15·85 | 11·38 | 14·68 | 13·79 |  |  |
| 20:0 Stigmasteryl ester/20:1 Sitosteryl ester_[M+K]^+^ | 745·626 | 0·53 | 1·04 | 0·27 | 2·35 | 2·78 | 2·96 | 4·39 |
| 20:0 Stigmasteryl ester/20:1 Sitosteryl ester_[M+NH_4_]^+^ | 724·6966 | 18·16 |  | 8·67 |  |  | 9·02 |  |
| 20:1 Campesteryl ester_[M+G]^+^ | 787·6721 |  |  |  | 0·17 | 5·24 | 6·50 | 14·26 |
| 20:1 Campesteryl ester_[M+K]^+^ | 731·6103 | 8·92 | 6·65 | 3·95 | 8·11 | 13·81 | 13·97 | 27·13 |
| 20:1 Stigmasteryl ester/20:2 Sitosteryl ester_[M+H]^+^ | 705·6549 |  | 31·68 | 36·35 | 34·94 | 24·83 | 30·13 | 35·67 |
| 20:1 Stigmasteryl ester/20:2 Sitosteryl ester_[M+K]^+^ | 743·6103 | 1·41 | 2·02 | 1·78 | 4·58 | 4·64 | 3·33 | 4·89 |
| 20:1 Stigmasteryl ester/20:2 Sitosteryl ester_[M+Na]^+^ | 727·6369 | 39·92 | 33·40 | 23·08 | 25·97 | 28·27 | 27·90 | 34·33 |
| 20:1 Stigmasteryl ester/20:2 Sitosteryl ester_[M+NH_4_]^+^ | 722·6809 | 4·82 | 4·78 | 3·05 | 3·59 | 3·39 | 3·16 | 4·26 |
| 20:2 Campesteryl ester_[M+H]^+^ | 691·6393 | 1·34 | 2·01 | 1·92 | 1·40 |  | 0·77 |  |
| 20:2 Campesteryl ester_[M+K]^+^ | 729·5947 | 16·68 | 11·85 | 9·27 | 10·83 | 16·79 | 18·61 | 25·32 |
| 20:2 Campesteryl ester_[M+Na]^+^ | 713·6213 | 0·50 | 0·71 |  |  | 1·29 | 0·77 |  |
| 20:2 Stigmasteryl ester/20:3 Sitosteryl ester_[M+G]^+^ | 797·6565 | 6·03 | 5·97 | 8·56 | 7·97 | 4·08 | 2·41 | 0·97 |
| 20:2 Stigmasteryl ester/20:3 Sitosteryl ester_[M+H]^+^ | 703·6393 | 1·20 | 2·07 | 1·11 | 1·37 | 2·39 | 4·30 | 3·06 |
| 20:2 Stigmasteryl ester/20:3 Sitosteryl ester_[M+K]^+^ | 741·5947 | 3·81 | 4·47 | 5·81 | 9·54 | 7·53 | 4·85 | 4·03 |
| 20:2 Stigmasteryl ester/20:3 Sitosteryl ester_[M+Na]^+^ | 725·6213 | 17·48 | 11·90 | 9·94 | 8·98 | 11·47 |  |  |
| 20:3 Campesteryl ester_[M+G]^+^ | 783·6408 | 19·92 | 32·45 | 36·39 | 69·63 | 71·49 | 58·70 | 50·51 |
| 20:3 Campesteryl ester_[M+K]^+^ | 727·579 | 32·65 | 24·84 | 11·72 | 4·55 | 19·98 | 25·68 | 29·65 |
| 20:3 Campesteryl ester_[M+NH_4_]^+^ | 706·6496 | 19·01 | 19·81 | 19·40 | 31·84 | 20·37 | 20·31 | 29·90 |
| 20:3 Stigmasteryl ester_[M+H]^+^ | 701·6236 | 1·56 | 1·20 | 1·38 | 1·10 | 2·56 | 2·57 | 1·20 |
| 20:3 Stigmasteryl ester_[M+K]^+^ | 739·579 | 6·15 | 7·40 | 7·77 | 15·34 | 14·95 |  | 13·74 |
| 20:3 Stigmasteryl ester_[M+Na]^+^ | 723·6056 |  | 5·80 | 3·55 | 2·04 | 2·61 | 3·18 | 7·11 |
| 22:0 Sitosteryl ester_[M+NH_4_]^+^ | 754·7435 | 62·85 | 54·09 | 50·74 | 59·35 | 43·05 | 44·17 | 43·23 |
| 22:0 Stigmasteryl ester_[M+G]^+^ | 829·7191 | 1·62 | 1·39 | 0·44 | 0·91 | 0·70 | 0·50 | 0·66 |
| 22:0 Stigmasteryl ester_[M+H]^+^ | 735·7019 | 65·11 | 84·93 | 63·88 |  | 100·13 | 69·86 |  |
| 22:0 Stigmasteryl ester_[M+NH_4_]^+^ | 752·7279 | 22·42 | 19·46 | 9·61 | 11·38 | 9·05 | 13·10 | 21·73 |
| 22:1 Campesteryl ester_[M+K]^+^ | 759·6416 | 17·50 | 20·25 | 16·21 | 45·15 | 49·76 | 44·73 | 51·64 |
| 22:1 Campesteryl ester_[M+NH_4_]^+^ | 738·7122 | 9·51 | 13·72 | 13·69 | 26·03 | 34·28 |  | 31·21 |
| 22:1 Stigmasteryl ester/22:2 Sitosteryl ester_[M+K]^+^ | 771·6416 | 12·93 | 13·64 | 14·79 | 22·41 | 16·85 | 14·63 | 7·06 |
| 22:2 Campesteryl ester_[M+G]^+^ | 813·6878 | 0·20 | 2·29 |  | 0·69 | 0·39 | 0·67 | 0·30 |
| 22:2 Campesteryl ester_[M+H]^+^ | 719·6706 | 3·23 | 3·68 | 2·29 | 2·58 | 3·25 | 2·21 | 4·00 |
| 22:2 Campesteryl ester_[M+K]^+^ | 757·626 | 49·87 | 47·98 | 46·51 | 58·76 | 56·76 | 49·07 | 54·41 |
| 22:2 Stigmasteryl ester/22:3 Sitosteryl ester_[M+H]^+^ | 731·6706 | 8·51 | 7·05 | 4·24 | 6·32 | 15·02 | 14·31 | 26·87 |
| 22:2 Stigmasteryl ester/22:3 Sitosteryl ester_[M+K]^+^ | 769·626 | 36·45 | 34·54 | 41·58 | 43·84 | 32·29 | 28·60 | 12·39 |
| 22:3 Campesteryl ester_[M+H]^+^ | 717·6549 | 1·49 | 3·06 | 0·43 | 0·42 | 2·03 | 2·13 | 0·89 |
| 22:3 Campesteryl ester_[M+K]^+^ | 755·6103 | 68·07 | 48·43 | 42·26 | 38·77 | 39·58 | 40·54 | 53·82 |
| 22:3 Campesteryl ester_[M+NH_4_]^+^ | 734·6809 | 101·47 | 116·48 | 145·09 | 139·22 | 181·99 | 143·88 | 189·69 |
| 22:3 Stigmasteryl ester_[M+H]^+^ | 729·6549 | 16·09 | 11·35 | 9·13 | 10·41 | 16·49 | 18·48 | 24·16 |
| 22:3 Stigmasteryl ester_[M+K]^+^ | 767·6103 | 46·18 | 48·02 | 66·95 | 66·57 | 70·46 | 45·86 | 19·42 |
| 22:3 Stigmasteryl ester_[M+NH_4_]^+^ | 746·6809 | 5·34 | 6·98 | 6·11 | 6·79 |  | 6·63 |  |
| Sitosteryl glucoside_[M+H]^+^ | 577·4468 | 10·19 | 9·80 | 7·42 | 16·87 | 20·20 | 17·00 | 20·66 |
| Sitosteryl glucoside_[M+K]^+^ | 615·4022 | 0·99 | 1·18 | 0·99 | 1·25 | 0·89 | 0·55 | 0·73 |
| Sitosteryl glucoside_[M+NH_4_]^+^ | 594·4728 | 1·07 | 0·73 | 0·62 | 0·60 | 0·74 | 0·71 | 0·81 |
| Sphinganine-1-phosphocholine_[M+G]^+^ | 561·3786 | 1·23 | 1·28 | 2·26 | 2·43 | 1·72 | 1·31 | 0·81 |
| Sphingosine-1-phosphate_[M+G]^+^ | 474·2738 | 0·56 | 0·59 | 0·65 |  | 0·78 |  | 1·00 |
| Stigmasteryl glucoside_[M+H]^+^ | 575·4311 | 5·55 | 6·99 | 5·70 | 12·70 | 14·52 | 10·12 | 11·97 |

*Table S6. Relative signal intensities for high resolution* m/z *values consistent with sterol isoforms (n = 2). Signals were assigned according to high resolution masses with a deviation of <12 ppm from the calculated monoisotopic mass. Quantitative standards were not used. G, guanidinium. Collection points: +2 h, early part of G_1_; +4 h, mid-G_1_ (first CP); +6 h, end of G_1_ and pS; +8 h, S and second CP; +10 h, G_2_; +13 h, M; +15 h, G_3_, for others see* Figure 1*.*

| **Primer** | **Sequence** |
| --- | --- |
| DqPIS1 F | AGCCCTTGCCAGTTGTCCAC |
| DqPIS1 R | GGCAGCAGCACACACAAGGT |
| DqCDKA F | GGCACGTATGGCGTGGTGTA |
| DqCDKA R | GTACAACCGCCGGTCAGCAT |
| DqCDKB F | AAAATCGGCGAGGGCACATA |
| DqCDKB R | CAGGCAGGGCTTGTCGTTCT |
| DqEPT1 F | AAGCGGAACAGCCGTGTAAC |
| DqEPT1 R | GTCCCCAGCAGCGTAATCAG |

*Table S7. Sequences of primers used for qPCR amplification of homologues of cell cycle and lipid pathway genes. Each primer set was spanning at least one intron and amplified an amplicon of 150-200 nt.*
